# Examining the gray cube effect on naïve viewers’ appreciation of street-based art in Hong Kong and Poland

**DOI:** 10.1038/s41598-024-53322-7

**Published:** 2024-02-19

**Authors:** Magdalena Szubielska, Robbie Ho, Anna Witeska-Młynarczyk, Natalia Kopiś-Posiej

**Affiliations:** 1 Institute of Psychology, The John Paul II Catholic University of Lublin, Lublin, Poland; 2 Department of Cultural and Creative Arts, The Education University of Hong Kong, Ting Kok, Hong Kong; 3 Institute of Sociology, Maria Curie-Skłodowska University in Lublin, Lublin, Poland; 4 Department of Clinical Neuropsychiatry, Faculty of Medicine, Medical University of Lublin, Lublin, Poland

**Keywords:** Psychology, Human behaviour

## Abstract

The present research investigates the appreciation of sanctioned street-based art among naïve viewers. It examines the role of viewing context in art appreciation, by experimentally testing a *gray cube effect*, which posits that street-based artworks are more likely to be identified as art (H1), liked more (H2), and understood more (H3) when viewed on the street. Identical procedures were carried out in Hong Kong (Experiment 1) and Lublin, Poland (Experiment 2), separately, sampling local artworks and local viewers. Experiment 1 tested 14 murals with 100 Hong Kongers; Experiment 2 tested 7 sculptures and 7 murals with 88 Poles. Participants were randomly assigned to either viewing street-based artworks on the street (gray cube) or viewing digital images of street-based artworks in a laboratory. The participants assessed each artwork in terms of art identification, liking, and understanding. These “twin” experiments yielded identical results, i.e., street-based artworks were liked more (H2) and understood more (H3) but not more likely to be identified as art (H1) on the street than in the laboratory. Overall, the present findings support the gray cube effect with ecologically valid data, and the effect seems robust across Western and Eastern cultural contexts and across genres of sculpture and mural.

## Introduction

Nowadays, it is increasingly common to encounter artistic creations on the street. This phenomenon began with an illegitimate urban subculture generally called street art, where artworks are created and displayed in public places without authorization as an attempt to challenge or critique existing social structures or societal issues. Part of this subculture underwent absorption into grander institutional schemes of urban development managed by cities worldwide interested in (re)branding themselves as creative metropolises through presenting artworks publicly^1^. This global strategy has incidentally raised the visibility and social acceptance of street art and similar acts such as graffiti, blurring the general perception and understanding of this once subversive art form. Artworks on the street are now often debatable on whether they should be seen as art or crime, public or private expression, ephemeral or permanent resident, and cultural or economic driver^2^. Departing from the sociological discussion, the present research adopts a psychological view to examine how viewers perceive and experience the sanctioned, “legal” form of artworks on the street, i.e., *street-based art/artworks*. Specifically, the present work investigates the role of the street (or *gray cube*, as explained later), as a viewing context, in enhancing the appreciation of street-based art.

### Definition of street art and street-based art

Street art is distinguished from institutionally approved public art as “unofficial” or “unsanctioned”^3^. Graffiti, for instance, are historically associated with vandalism and criminal acts^4^. The “illegal” component of street art and graffiti can be seen as a critique of capitalism and consumerism, which challenges the existing system of public art that tends to restrict artistic freedom and instead align artworks with commercial interests. Thus, street art can be a political means against a consumer-driven art world^5,6^. While it is tempting to quickly adopt the binary terms of “legal” and “illegal” for defining street-based art versus street art, such an approach could still be problematic. Although works originating from an illegitimate subculture are usually minimally recognized by the mainstream art world, it is now common that such works become acknowledged and reappropriated as valuable by established museums, galleries, collectors, and auction houses (e.g., the case of *Banksy’s Slave Labour*;^7^). Likewise, private owners may attempt to protect illegal street art (e.g., by shielding artworks with *Perspex*;^8^). Furthermore, the so-called process of “artification”, i.e., transforming ordinary urban spaces into artistic and creative environments, has further enhanced the visibility and social acceptance of street art and graffiti^9,10^. Under the scheme of artification, “illegal” street artists may now be invited to collaborate with local communities officially. Overall, for reasons related to politics of art and schemes of urban development, the once subversive art form has transformed into a more visible and socially accepted phenomenon in major cities worldwide. These days, there are walking tours of street art backed up by local councils or private developers^11^. Historical sites featuring street art have even become touristic attractions for some^12^. Given its artistic and creative nature, street art may lift the spirit of a city—the *genius loci* in Lewicka’s words^13^—by making public places more interesting and more pleasant to look at. Empirical data support that street entertainments can enhance people’s perception of public places^14–17^.

To avoid definitional ambiguity that comes with “street art”, this article cautiously adopts the seemingly neutral term “street-based art” to refer to the artworks being examined in the present research. Street-based art may take various forms such as murals painted on public walls or sculptures and installations sited in public places such as squares or plazas^18,19^. The street-based artworks considered in the present research appeared in public places legally and were a part of the planned artification sanctioned and financed by local authorities and administrations devoted to urban growth.

### A psychological perspective on the appreciation of street-based art

Psychological research related to the perception and experience of artworks on the street has grown over the years. For example, Mitschke et al.’s^19^ eye-tracking study showed that their participants spent up to 50% of the total fixation time looking at graffiti and sculptures in a naturalistic setting. Recently, researchers have compared street-based artworks against non-street-based artworks in terms of viewers’ experience. For instance, Chamberlain et al.^18^ found that the attractiveness ratings of graffiti tags and murals tended to be lower than those of representational and abstract paintings. In the same vein, Szubielska and Ho^20^ found that murals were less likely to be seen as art than both representational and abstract paintings, and that murals were liked less than representational paintings but more than abstract paintings.

The present research focuses on the *appreciation of street-based art (art appreciation)* among *naïve viewers*, i.e., people without previous formal or informal training in art practice or art history. In line with Ho et al.^21^ and Wang and Ishizaki^22^, the present work operationalizes art appreciation as *art identification*, *liking*, and *understanding*. Art appreciation may be associated with art viewers’ (a) prior exposure to artworks, (b) expertise and interest in art, and (c) personality trait of need for closure (NFC). First, aesthetic judgements may be attributed to people’s familiarity with certain artworks^23^. The mere-exposure effect suggests that people’s preference for a given stimulus increases as a function of repeated exposure to that stimulus^24^. Thus, the more familiar people feel about certain artworks, the more favorably they will perceive the artworks. Second, art appreciation may be influenced by people’s expertise in art—people’s knowledge base about art they have acquired through formal and/or informal experiences^23^. People with high art expertise tend to appreciate artworks more than people with low art expertise do^25–29^. Therefore, people’s expertise and interest in art should be controlled for when investigating art appreciation. Third, art appreciation may be affected by the personality trait of NFC—desire for predictability, preference for order and structure, and discomfort with ambiguity^30^. People high in NFC tend *not* to favor non-realistic, abstract, and ambiguous artworks^31–34^. Unlike traditional artworks (e.g., classical paintings), street-based art often comprises patterns and imageries that are unpredictable, disorganized, and open-ended. Hence, people high in NFC may struggle to appreciate street-based art. Put together, the present research examines naïve viewers’ art identification, liking, and understanding of street-based art while being cautious of the potential confounding of familiarity, expertise and interest in art, and NFC.

### The gray cube effect on the appreciation of street-based art

The present work highlights the role of viewing context in determining viewer’s art appreciation. The assumption that art appreciation depends on viewing context is well documented in the realm of non-street-based artworks^35–39^. The significance of viewing context should also be expected in modelling the appreciation of street-based art. Gartus and Leder^40^ first coined “gray cube” to refer to the street context as opposed to “white cube” that refers to the museum or gallery context. The present research aims at verifying a *gray cube effect*—i.e., street-based artworks are appreciated more when they are viewed on the street. Street-based art is deliberately displayed on the street, and so the street should be the ideal viewing context for appreciating street-based artworks^41^. The street, as a viewing context, makes room for multisensorial explorations and aesthetic encounters characterized by serendipity, enriching the art reception and meaning-making processes among the viewers^3^.

Empirically, two experimental studies have tested the gray cube effect and yielded inconclusive findings^40,42^. Gartus and Leder^40^ compared the aesthetic experiences between graffiti artworks embedded in street scenes (gray cube) and those embedded in museum scenes (white cube). The main effect of viewing context was nonsignificant. The effect only manifested when viewing context interacted with the viewers’ interest in graffiti art—viewers high in graffiti interest reported higher aesthetic emotions for graffiti artworks embedded in street scenes. With a similar design, Gartus et al.^42^ obtained similar results on beauty ratings of graffiti. Again, the main effect of viewing context was nonsignificant, and the effect only manifested when viewing context and the viewers’ graffiti interest interacted—graffiti artworks embedded in street scenes received higher beauty ratings among viewers high in graffiti interest. In any case, the ecological validity of these studies could be limited, as they were only conducted in the laboratory where the participants were only shown digital images of street-based artworks without seeing the actual artworks on-site^40,42^. Hence, an ecologically valid verification of the gray cube effect that includes on-site viewing of actual street-based artworks has been due. The present work seeks to fill this gap.

### The present study

The present research aims at testing the gray cube effect on the appreciation of street-based art among naïve viewers. We set up experiments to compare viewers’ art appreciation between two viewing contexts: street versus laboratory. The street context refers to viewing street-based art on-site; the laboratory context refers to viewing digital images of street-based art in a laboratory. We hypothesize that *street-based artworks are more likely to be identified as art (H1), liked more (H2), and understood more (H3) when they are viewed on the street than when they are viewed in a laboratory*.

To test these hypotheses, we set up twin experiments in Hong Kong and Lublin, Poland. On the Asian side, Hong Kong, officially the *Hong Kong Special Administrative Region of the People’s Republic of China*, is located at the south-eastern tip of China and has a current population of approximately 7.35 million that comprises primarily people of Chinese descent^43^. According to Pan^44^, Hong Kong is among the first cities in the China region where street art and graffiti emerged before spreading to others such as Beijing and Shanghai. To date, street-based art remains common in Hong Kong^45^. On the European side, Lublin is located in the eastern part of Poland. With approximately 340,000 inhabitants, it is the ninth largest city in the country population-wise. Along with the advancing democratization after the collapse of the socialist regime, Lublin has embraced street art and graffiti as part of its new image. Thanks to a vibrant urban art community, street-based art is promoted through international festivals such as *Meeting of Styles*.

To clarify, our experiments are *not* intended as a cross-cultural comparison on the gray cube effect or appreciation of street-based art. Instead, they serve to test the robustness of the effect in various cultural contexts. While previous experiments examined the effect solely in a European context^40,42^, the present work broadens the investigation to an Asian context to extend generalizability of research findings.

The present work was approved by the Ethical Committee of the Institute of Psychology of The John Paul II Catholic University of Lublin and was conducted in accordance with the Declaration of Helsinki.

## Experiment 1: the gray cube effect in Hong Kong

### Method

#### Participants

Sample size was determined based on previous studies on the influence of physical viewing context on art appreciation^38,39^. An a priori power analysis using G*Power 3.1^46^ suggested that, to achieve a power of 0.95 for detecting the influence of physical context on aesthetic preference with an effect size of *f* = 0.44^39^ at a significance level of *p* < 0.05, *N* = 72 participants would be needed for a between-subjects effect of viewing context in an analysis of variance (ANOVA). Due to possible individual differences in aesthetic experience among naïve viewers, we sought to recruit more than 72 participants (for each experiment).

Participants were 100 students (66 women and 1 preferred not to report gender) aged between 17 and 29 years (*M* = 18.59, *SD* = 1.68; 4 preferred not to report age). All were Hong Kongers and were recruited by the Research Team. All participants were naïve viewers; they declared having no previous formal or informal training in art practice or art history. The participants were randomly assigned to one of four conditions that were formulated with two *viewing contexts* (street vs. laboratory) and two *viewing orders* (artworks 1–14 vs. artworks 14–1): (a) street viewing of artworks 1–14 (*n* = 23; 17 women, 1 preferred not to report gender; *M*_age_ = 18.27 years, *SD* = 0.77); (b) street viewing of artworks 14–1 (*n* = 25; 18 women; *M*_age_ = 18.54 years, *SD* = 1.35); (c) laboratory viewing of artworks 1–14 (*n* = 26; 17 women; *M*_age_ = 18.77 years, *SD* = 1.66); and (d) laboratory viewing of artworks 14–1 (*n* = 26; 14 women; *M*_age_ = 18.75 years, *SD* = 2.49). The participants received 70 Hong Kong dollars per person as an incentive. Written informed consent was obtained from all participants before data collection.

#### Stimuli

Stimuli were 14 street-based murals (artworks 1–14) located around the *SoHo district* in Hong Kong (see Supplementary Material S1). The area was characterized as an entertainment zone with bars and restaurants. The total walking distance from artworks 1 through 14 was about 1.0 km. Participants in the street conditions were shown the actual artworks on-site; those in the laboratory conditions were shown digital images of the artworks.

#### Procedure and materials

In the street conditions, the participants, in groups of about five people, took a walking tour with an experimenter to view and assess the stimuli on-site. The tours took place during the day when the artworks could be seen under sunlight. The meeting point was away from any of the artworks to prevent the participants from seeing any of them while receiving study instructions. In the laboratory conditions, the participants sat individually in a laboratory to view and assess the stimuli on a 22-inch computer screen with 1920 × 1080 resolution. In both conditions, the artworks were processed one by one, either in the order of 1–14 or 14–1. The manipulation of viewing order is merely operational. Given the street element of this experiment, it was unrealistic to fully randomize the viewing order. The best we could do was to flip the order completely (i.e., 1–14 vs. 14–1) for half of the participants.

Upon viewing each artwork, the participants assessed it in terms of *art identification* (“*How much do you feel that this work is art?*”), *liking* (“*How much do you like this work?*”), *understanding* (“*How much do you feel that you have understood this work?*”), and *familiarity* (“*How much does this work feel familiar to you?*”) on a 7-point Likert scale (from 1 = *not at all* to 7 = *extremely*). After assessing all 14 artworks, the participants answered four questions about their expertise and interest in art, on the same 7-point scale: *expertise in the visual arts* (“*How much do you consider yourself an expert in the visual arts?*”), *interest in the visual arts* (“*How much are you interested in the visual arts?*”), *expertise in contemporary art* (“*How much do you consider yourself an expert in contemporary art?*”), and *interest in contemporary art* (“*How much are you interested in contemporary art?*”). Finally, they completed 15 items of the *Need for Closure Scale* short version, on a 6-point scale (from 1 = *strongly disagree* to 6 = *strongly agree*)^30,47^.

In all conditions, the participants were instructed not to talk or discuss their answers with others during the study. There were no time limits nor breaks during the studies. The entire procedure took about 60 and 30 min on the street and in the laboratory, respectively.

### Results

#### Preliminary analyses

Preliminary analyses first examined whether the four experimental groups differed in their NFC, expertise and interest in art, and familiarity with the selected artworks (for details, see Supplementary Material S2). Although NFC was higher among the street groups than the laboratory groups, it did not correlate with art identification (*r* = 0.03, *p* = 0.782), liking (*r* = 0.06, *p* = 0.557), nor understanding (*r* = 0.06, *p* = 0.546). The four groups did not differ in their expertise nor interest in art. Regarding familiarity, we observed a significant main effect of viewing context which in turn interacted with viewing order. Overall, familiarity was higher among the street groups than the laboratory groups. Such a difference could be due to a broader viewing perspective afforded by the street context in comparison with the laboratory context. In the laboratory, artworks were experienced through viewing photographs that had well-defined borders limiting the information the viewers could receive. On the street, contrarily, artworks were experienced through viewing on-site where an infinite and unpredictable amount of extra information could reach the viewers. Such extra information might include stimuli familiar to the viewers, such as familiar buildings, shopfronts, pedestrians, etc. Viewing artworks under such familiar surroundings, it makes sense that the artworks could also be experienced as more familiar. Further, we tested whether NFC and expertise and interest in art could predict art appreciation. Overall, both art identification and liking could be predicted by expertise and interest in contemporary art, while understanding could only be predicted by interest in contemporary art.

#### Main analyses

**Figure 1:**
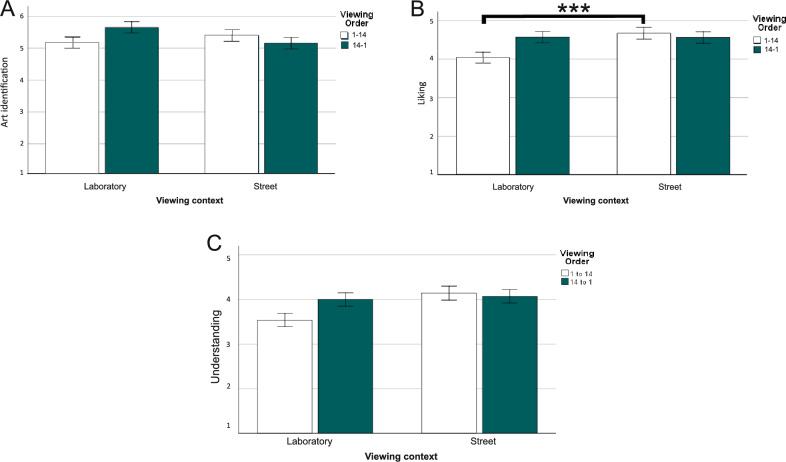
Post hoc comparisons of the significant interaction effect of *viewing context* and *viewing order* on *art identification* (panel **A**), *liking* (panel **B**), and *understanding* (panel **C**). Error bars represent ± 1 standard error. **p < 0.01.

Descriptive statistics of art identification, liking, and understanding are presented in Table 1. To test the hypotheses regarding the gray cube effect, we conducted three ANOVAs for art identification (H1), liking (H2), and understanding (H3), respectively, with viewing context (street vs. laboratory) and viewing order (1–14 vs. 14–1) as between-subjects factors. Complete results are presented in Table 2 (see also Fig. 1A–C).

**Table 1:** Art identification, liking, and understanding of murals as a function of viewing context and viewing order (experiment 1).

	Street		Laboratory	
	Order 1–14	Order 14–1	Order 1–14	Order 14–1
	*M* (*SD*)	*M* (*SD*)	*M* (*SD*)	*M* (*SD*)
Art identification	5.39 (1.01)	5.15 (0.78)	5.16 (0.90)	5.65 (0.90)
Liking	4.66 (0.69)	4.56 (0.59)	4.03 (0.84)	4.57 (0.83)
Understanding	4.15 (0.71)	4.07 (0.67)	3.54 (0.78)	4.00 (0.85)

**Table 2:** ANOVA effects of viewing context and viewing order on art identification, liking, and understanding (experiment 1).

Effect	Art identification			Liking			Understanding		
	*F*(1, 96)	*p*	η_p_^2^	*F*(1, 96)	*p*	η_p_^2^	*F*(1, 96)	*p*	η_p_^2^
Viewing context	0.54	0.463	0.01	**4.32**	**0.040**	**0.04**	**5.02**	**0.027**	**0.05**
Viewing order	0.52	0.471	0.01	2.07	0.153	0.02	1.68	0.198	0.02
Viewing context × viewing order	**4.02**	**0.048**	**0.04**	**4.57**	**0.035**	**0.05**	3.13	0.080	0.03

Significant effects are bold.

*Art Identification* The main effects of viewing context and viewing order were both nonsignificant. The interaction between viewing context and viewing order was significant, although post hoc comparisons (with Bonferroni adjustment, here and hereafter; see Fig. 1A) yielded no further significant results (all *p*s > 0.05).

*Liking* The main effect of viewing context was significant. Street-based murals were liked more on the street (*M* = 4.61, *SE* = 0.11) than in the laboratory (*M* = 4.30, *SE* = 0.10). This effect was qualified by a significant interaction with viewing order. Post hoc comparisons (see Fig. 1B) showed that the effect was significant only in the condition of viewing order 1–14 (*p* = 0.004). The main effect of viewing order was nonsignificant.

*Understanding* The main effect of viewing context was significant. Street-based murals were understood more on the street (*M* = 4.11, *SE* = 0.11) than in the laboratory (*M* = 3.77, *SE* = 0.11). The main effect of viewing order and the interaction between viewing context and viewing order were both nonsignificant.

### Discussion

Experiment 1 verified the gray cube effect with street-based murals located in Hong Kong and Hong Kong naïve viewers. The street-based murals were liked more and understood more when viewed on the street than in the laboratory. Hence, H2 and H3 are both supported. However, when it comes to liking, the effect interacted with viewing order and was only significant in the condition of order 1–14. Notably, a similar effect was observed in the control variable of familiarity (see Supplementary Material S2, Fig. S1). Hence, it is not impossible that greater familiarity had contributed to the effect on liking^24^. In terms of art identification, ratings did not differ significantly between the street and laboratory conditions. Hence, H1 is not supported. Overall speaking, Experiment 1 supports the gray cube effect.

The current experiment has two limitations. First, the current results could be confounded by a difference between group and individual viewings, since the street participants viewed the artworks collectively whereas the laboratory participants viewed the artworks individually. Art appreciation might be sensitive to social contexts. Field studies have shown behavioral differences between groups and individuals in viewing art. For example, museum visitors tend to spend more time viewing artworks when they are in large groups than when they are not^48,49^. Second, the current results might not generalize across different art genres, since only a single genre of street-based art underwent our examination. It is crucial to determine if the gray cube effect is robust across different genres of street-based art. These limitations will be addressed in Experiment 2.

## Experiment 2: the gray cube effect in Lublin

### Method

#### Participants

Sample size was determined similarly to Experiment 1. Participants were 88 students (61 women) aged between 19 and 34 years (*M* = 22.44, *SD* = 2.22). All were Poles who had lived in Lublin for at least one year and were recruited by the Research Team. All participants were naïve viewers who declared having no previous formal or informal training in art practice or art history. The participants were randomly assigned to one of four conditions that were formulated with two *viewing contexts* and two *viewing orders*: (a) street viewing of artworks 1–14 (*n* = 22; 13 women; *M*_age_ = 22.14 years, *SD* = 1.78); (b) street viewing of artworks 14–1 (*n* = 22; 12 women; *M*_age_ = 22.00 years, *SD* = 1.69); (c) laboratory viewing of artworks 1–14 (*n* = 22; 15 women; *M*_age_ = 22.41 years, *SD* = 1.53); and (d) laboratory viewing of artworks 14–1 (*n* = 22; 21 women; *M*_age_ = 23.23 years, *SD* = 3.34). The participants did not receive any incentives. Written informed consent was obtained from all participants before data collection.

#### Stimuli

Stimuli were seven street-based sculptures (artworks 1–7) and seven street-based murals (artworks 8–14) located around the *LSM housing estate* in Lublin (see Supplementary Material S3). The area was characterized by modernist architecture and green belts. The total walking distance from artworks 1 through 14 was about 1.2 km. Participants in the street conditions were shown the actual artworks on-site; those in the laboratory conditions were shown digital images of the artworks.

#### Procedure and materials

Procedure was similar to that of Experiment 1, except that both the street and laboratory participants were assigned to proceed in groups of about five people, i.e., collectively. Participants in the street conditions viewed and assessed the stimuli on-site; those in the laboratory conditions viewed and assessed the stimuli on a 63-inch projector screen with 1920 × 1080 resolution. Since the artworks were either processed in the order of 1–14 or 14–1, the participants either processed sculptures first or murals first.

Like Experiment 1, the participants assessed each artwork in terms of *art identification*, *liking*, *understanding*, and *familiarity*. For expertise and interest in art, the participants answered two questions about their *expertise in the visual arts* and *interest in contemporary art*. Finally, they completed 15 items of the *Need for Closure Scale* short version in Polish^50^; two participants did not complete this scale.

Like Experiment 1, the participants in all conditions were instructed not to talk or discuss with others during the study, and the entire procedure took about 60 and 30 min on the street and in the laboratory, respectively.

### Results

#### Preliminary analyses

Preliminary analyses similar to those of Experiment 1 were conducted (for details, see Supplementary Material S4). Although NFC differed across the four experimental groups, it did not correlate with art identification (*r* = 0.00, *p* = 0.992), liking (*r* =  − 0.04, *p* = 0.736), nor understanding (*r* =  − 0.03, *p* = 0.800). The four groups did not differ in their expertise nor interest in art. Regarding familiarity, we observed a significant main effect of viewing context which in turn interacted with viewing order. Overall, familiarity was higher among the street groups than the laboratory groups. Like Experiment 1, such a difference could be due to a broader viewing perspective afforded by the street context in comparison with the laboratory context. Further, both art identification and understanding could be predicted by interest in contemporary art, while liking could be predicted by expertise in the visual arts.

#### Main analyses

Descriptive statistics of art identification, liking, and understanding are presented in Table 3. In this experiment, genre of the selected artworks varied between sculpture and mural, and it was entangled with viewing order. To test the hypotheses regarding the gray cube effect, we conducted three ANOVAs for art identification (H1), liking (H2), and understanding (H3), respectively, with viewing context (street vs. laboratory) and viewing order (1–14/sculptures–murals vs. 14–1/murals–sculptures) as between-subjects factors and genre (sculpture vs. mural) as a within-subjects factor. Complete results are presented in Table 4.

**Table 3:** Art identification, liking, and understanding of sculptures and murals (split and collapsed) as a function of viewing context and viewing order (experiment 2).

	Street		Laboratory	
	Order 1–14 (Sculptures–Murals)	Order 14–1 (Murals–Sculptures)	Order 1–14 (Sculptures–Murals)	Order 14–1 (Murals–Sculptures)
	*M* (*SD*)	*M* (*SD*)	*M* (*SD*)	*M* (*SD*)
	*Sculptures*			
Art identification	3.98 (1.00)	4.42 (1.17)	3.89 (0.96)	4.32 (0.89)
Liking	3.94 (0.97)	4.73 (0.83)	3.51 (0.94)	4.60 (0.80)
Understanding	3.34 (0.94)	4.43 (0.94)	2.75 (0.68)	4.36 (0.80)
	*Murals*			
Art identification	5.00 (0.88)	4.06 (1.23)	4.68 (1.36)	3.82 (1.25)
Liking	5.32 (0.72)	3.45 (1.15)	4.66 (1.21)	3.12 (1.01)
Understanding	4.88 (0.94)	2.89 (1.15)	3.99 (1.11)	2.82 (0.88)
	*Sculptures and murals*			
Art identification	4.49 (0.86)	4.24 (1.06)	4.29 (0.99)	4.07 (0.87)
Liking	4.63 (0.79)	4.09 (0.80)	4.08 (0.94)	3.86 (0.79)
Understanding	4.11 (0.85)	3.66 (0.90)	3.37 (0.81)	3.59 (0.69)

**Table 4:** ANOVA effects of viewing context, viewing order, and genre on art identification, liking, and understanding (experiment 2).

Effect	Art identification			Liking			Understanding		
	*F*(1, 84)	*p*	η_p_^2^	*F*(1, 84)	*p*	η_p_^2^	*F*(1, 84)	*p*	η_p_^2^
Viewing context	0.86	0.358	0.01	**4.81**	**0.031**	**0.05**	**5.36**	**0.023**	**0.06**
Viewing order	1.32	0.253	0.02	**4.58**	**0.035**	**0.05**	0.43	0.515	0.01
Genre	3.77	0.056	0.04	0.32	0.573	0.00	0.51	0.479	0.01
Viewing context × viewing order	0.01	0.930	0.00	0.83	0.366	0.01	3.70	0.058	0.04
Viewing context × genre	0.58	0.447	0.01	0.99	0.323	0.01	0.51	0.479	0.01
Viewing order × genre	**30.59**	**0.000**	**0.27**	**155.43**	**0.000**	**0.65**	**212.40**	**0.000**	**0.72**
Viewing context × viewing order × genre	0.03	0.862	0.00	0.01	0.939	0.00	0.55	0.459	0.01

Significant effects are bold.

**Figure 2:**
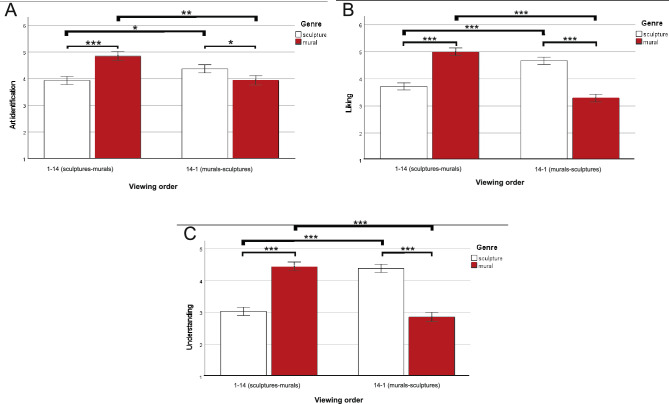
Post hoc comparisons of the significant interaction effect of *viewing order* and *genre* on *art identification* (panel **A**), *liking* (panel **B**), and *understanding* (panel **C**). Error bars represent ± 1 standard error. *p < 0.05, **p < 0.01, ***p < 0.001.

*Art Identification* The main effects of viewing context and viewing order and their interaction were all nonsignificant. The interaction between viewing order and genre was significant. Post hoc comparisons (see Fig. 2A) showed that, sculptures were more likely to be identified as art than murals when they were viewed after murals (*p* = 0.013), while murals were more likely to be identified as art than sculptures when they were viewed after sculptures (*p* < 0.001). Moreover, sculptures were more likely to be identified as art when viewed in 14–1 order than in 1–14 order (*p* = 0.045), and murals were more likely to be identified as art when viewed in 1–14 order than in 14–1 order (*p* < 0.001).

*Liking* The main effect of viewing context was significant. Street-based artworks were liked more on the street (*M* = 4.36, *SE* = 0.13) than in the laboratory (*M* = 3.97, *SE* = 0.13). The main effect of viewing order was significant, and it was qualified by a significant interaction with genre. Post hoc comparisons (see Fig. 2B) showed that sculptures were liked more than murals when they were viewed after murals, while murals were liked more than sculptures when they were viewed after sculptures (both *p*s < 0.001). Moreover, sculptures were liked more when viewed in 14–1 order than in 1–14 order, and murals were liked more when viewed in 1–14 order than in 14–1 order (both *p*s < 0.001).

*Understanding* The main effect of viewing context was significant. Street-based artworks were understood more on the street (*M* = 3.88, *SE* = 0.12) than in the laboratory (*M* = 3.48, *SE* = 0.12). The main effect of viewing order was nonsignificant. The interaction between viewing order and genre was significant. Post hoc comparisons (see Fig. 2C) showed that sculptures were understood more than murals when they were viewed after murals, while murals were understood more than sculptures when they were viewed after sculptures (both *p*s < 0.001). Moreover, sculptures were understood more when viewed in 14–1 order than in 1–14 order, and murals were understood more when viewed in 1–14 order than in 14–1 order (both *p*s < 0.001).

### Discussion

Experiment 2 replicated the gray cube effect with street-based sculptures and murals located in Lublin and Polish naïve viewers. The street-based artworks were liked more and understood more when viewed on the street than in the laboratory. Hence, like Experiment 1, H2 and H3 are both supported. In terms of art identification, also like Experiment 1, ratings did not differ significantly between the street and laboratory conditions. Hence, H1 is again not supported. Additionally, an unexpected interaction between viewing order and genre was observed. Sculptures received greater appreciation when they were viewed after murals, while murals received greater appreciation when they were viewed after sculptures. Thus, a given genre of street-based artworks was appreciated more favorably when it was viewed after the viewing of a different genre. These patterns were true in art identification, liking, and understanding. This finding contradicts the art fatigue effect which suggests that art appreciation decreases as a function of repeated art viewing^51^. Hence, a genre change might alleviate art fatigue. Further studies may clarify this contradiction by testing the impact of genre change in moderating the art fatigue effect. In any case, genre did not moderate the gray cube effect. Overall speaking, Experiment 2 further supports the gray cube effect.

## General discussion

### Summary of the present research and key findings

The gray cube effect posits that street-based artworks are appreciated more favorably when they are viewed on the street. To empirically test this effect, twin experiments were carried out in Hong Kong (Experiment 1) and Lublin, Poland (Experiment 2), to compare naïve viewers’ art identification (H1), liking (H2), and understanding (H3) of street-based artworks between two viewing contexts: street versus laboratory. Overall, findings of these experiments support the effect. In both experiments, street-based artworks were liked more (H2) and understood more (H3) on the street. This agrees with the assumption that the street, as a viewing context, serves as an optimal “exhibition space” for street-based art^41^. These results not only confirm the previously inconclusive findings^40,42^, but they also highlight the importance of viewing context to art appreciation in the broader context of empirical aesthetics^35–39^.

Nonetheless, the current findings do not support the effect in art identification (H1). Thus, viewing context might not influence art identification. It is plausible that artistic style is a more critical factor than viewing context. For example, past research has found that traditional paintings are more likely to be identified as art than modern and contemporary paintings^52^. Also, both traditional and modern paintings are more likely to be identified as art than murals^20^. To follow up this issue, future studies may incorporate both factors of artistic style and viewing context in modelling the appreciation of street-based art.

### Contributions

The present research has several notable contributions to the literature. First, to the best of our knowledge, this is the first experimental research to empirically test the gray cube effect in an ecologically valid setting, i.e., the street. Extant studies on the gray cube effect have been limited to the laboratory setting, where only digital images of street-based artworks were assessed without a true comparison with the actual artworks on-site^40,42^. Hence, the present work contributes ecologically valid findings regarding the gray cube effect.

Second, the present research includes artworks and viewers from different cultures, i.e., Hong Kong and Poland. Research on the gray cube effect^40,42^ and street-based art in general^18–20^ has been largely based on Western populations. By including an Asian population, this works moves beyond the W aspect of “WEIRD” (i.e., Western, Educated, Industrialized, Rich, and Democratic^53^) and thereby enhances the external validity of research findings related to the appreciation of street-based art.

Last but not least, the present research considers different genres of street-based sculptures and murals (Experiment 2). Generally, there is little research on the aesthetic experience of sculpture. A field study in the Austrian Gallery Belvedere found that sculptures were less preferred than paintings^54^. However, interestingly, the present findings suggest that sculptures and murals could be equally appreciated, and that genre difference in art appreciation could depend on viewing order. Although further research is needed to clarify this genre × order interaction, the present findings contribute to the literature on empirical aesthetics toward sculpture.

### Limitations

The present research has certain limitations. First, the gray cube effect could be entangled with a *genuineness effect*—genuine artworks may provide more intense viewing experiences in comparison with reproductions of artworks (for reviews, see^37,55^). In the present work, the participants either viewed genuine street-based artworks on-site or digital reproductions on a screen. Therefore, we remain uncertain if the greater art appreciation found on the street was really due to the gray cube or the genuineness of the artworks. Similar concerns have been raised in studies about artworks in the museum/gallery^35,36,38,39^. A recent meta-analysis focusing on the genuineness effect found a small effect, although the effect appeared confounded with viewing context^55^. Among studies without the confound of viewing context, the genuineness effect seemed to disappear. However, the researchers warn against drawing a strong conclusion too soon, as only a small number of studies (*n* = 3) have examined the genuineness effect without the confound of viewing context^55^. Experimental design controlling both viewing context and genuineness of artworks will be necessary to clarify the gray cube effect. Such a design has been carried out in a laboratory setting^40^, and it will be worth replicating in a field or street setting in the future. While it may be unrealistic to reproduce existing street-based artworks of comparable genuineness level in the laboratory, it is possible to create novel, believable stimuli that can be reproduced identically genuinely across the street and the laboratory. Thus, the gray cube effect could still be valid, although a closer examination teasing out the genuineness effect is required before concluding the gray cube effect.

Second, the viewing perspective in assessing street-based artworks could differ between the street and laboratory conditions. On the street, the participants might spontaneously engage with the artworks from various angles and distances, as they were viewing on-site. In the laboratory, however, the participants could only engage with the artworks from uniform angles and distances, as they were viewing on a screen. Street-based art may lose expressiveness when abstracted from the street context which supposedly provides a rich resource for creative dynamics and meaning-making processes to happen^56^. As a consequence, the possible interactivity with artworks could be higher on the street than in the laboratory, and thereby contributed to the gray cube effect. However, the findings on the influence of interactivity on art appreciation have been mixed. While some have found that interactivity could boost art appreciation^57,58^, others have found that interactivity could lose effect when entangled with the physical context^59^, as in the case of the current study. In future investigations, these variables may be controlled for (e.g., through using virtual reality in a laboratory).

Third, despite that the present work employed artworks and viewers from different cultures, we did not pursue a cross-cultural comparison nor theorize any cultural variables. Thus, at a cultural level, the current study only represents a basic attempt on assessing the gray cube effect (or appreciation of street-based art in general) in various cultural contexts. A lack of a theoretical cultural component limits the potential to further integrate the findings of the two experiments. A cultural framework would be required to explore the cross-cultural meanings regarding the gray cube effect. For example, *cultural match* between artworks and viewers can influence art appreciation^21,60,61^. The present findings assume cultural match (i.e., Hong Kong artworks matched with Hong Kong viewers and Polish artworks matched with Polish viewers) and so it remains unanswered whether culturally matching versus mismatching street-based artworks could be appreciated differently. It has been suggested that Hong Kongers (lower) and Poles (higher) differ in the trait of *uncertainty avoidance*^62^. Hence, while Hong Kongers might be equally receptive to culturally matching and mismatching artworks due to low uncertainty avoidance, Poles might be more receptive to matching but less so to mismatching artworks due to high avoidance. Understandably, the present study could not assign the participants to the different geographic location (i.e., Hong Kongers to Poland and Poles to Hong Kong), although such a design would have been more impactful by examining this cultural hypothesis while increasing the ecological validity of the findings. Future research may consider a laboratory setting where viewing context, cultural group, and cultural context can be controlled at the same time. While a laboratory setting would be one step away from the field, it will allow investigation into the cultural mechanisms that could be at play in the gray cube effect.

Fourth, expertise and interest in art were self-reported, and so related findings should be interpreted with caution. Due to a potential social approval factor, participants could have overestimated their expertise and interest in art. A more systematic and validated measurement would be the Vienna Art Interest and Art Knowledge questionnaire (VAIAK;^63^). However, VAIAK is Western-focused and neither a Hong Kong nor Polish adaptation has been developed. In any case, all participants in the present research declared having no previous formal or informal training in art practice or art history at the point of recruitment, and their self-reported ratings of art expertise were generally below the scale midpoint, suggesting that they were naïve viewers with little knowledge of art.

Finally, the present studies did not control for the viewers’ familiarity with the locations where the selected artworks were exhibited. Familiarity with artwork location may interact with the gray cube effect, in the sense that familiar versus unfamiliar feelings (e.g., novelty) about a location may influence the appreciation of the artworks under question. Consideration of this aspect of the viewing experience will enrich our understanding about the psychological processes of appreciating street-based art.

### Supplementary Information

Supplementary Information.

## Data Availability

The data that support the findings are openly available^64^: https://osf.io/p26f3/?view_only=1e1e5e2a5416429aabcf977e186be31f.
