# Supplementary material for: Examining the gray cube effect on naïve viewers’ appreciation of street-based art in Hong Kong and Poland
Source: Sci Rep. 2024 Feb 19;14:4099. doi: 10.1038/s41598-024-53322-7 (PMC10876577; doi:10.1038/s41598-024-53322-7)
Supplement: Supplementary file 1 — Supplementary Information. [file 41598_2024_53322_MOESM1_ESM.pdf]

## Supplementary Material 1

## Digital Reproductions of Artworks Used in Experiment 1

The numbering of the artworks corresponds to the order in which they were shown to the participants.

Artwork 1

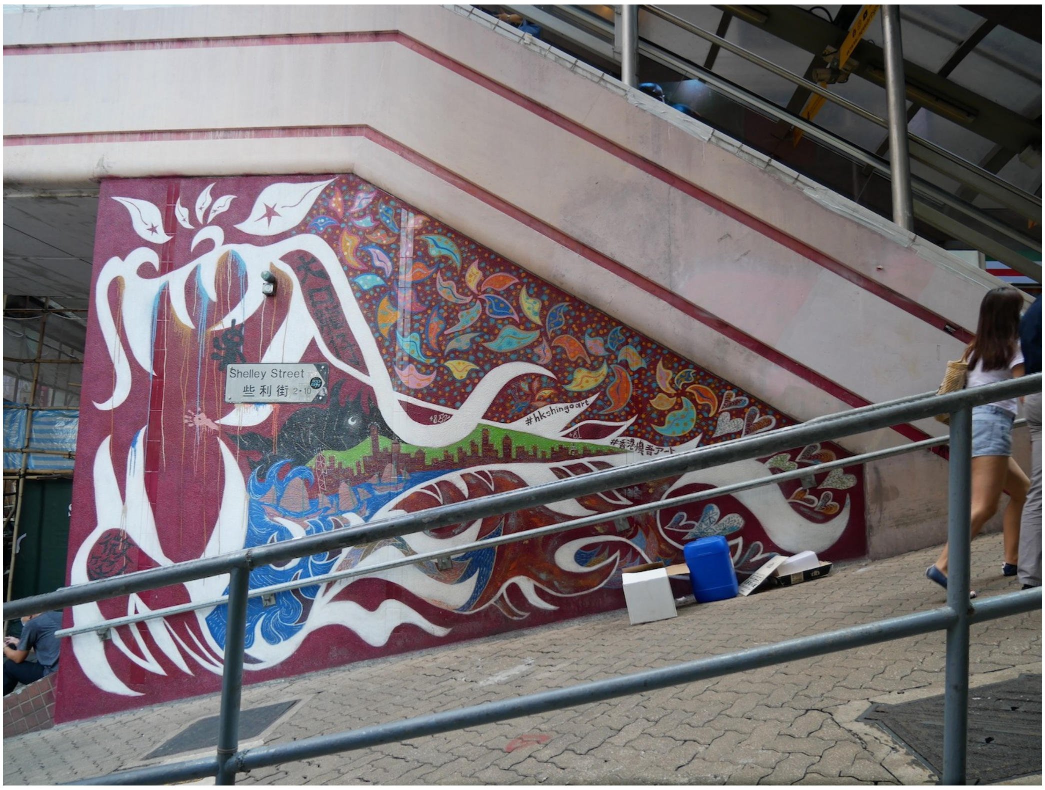


Artwork 2

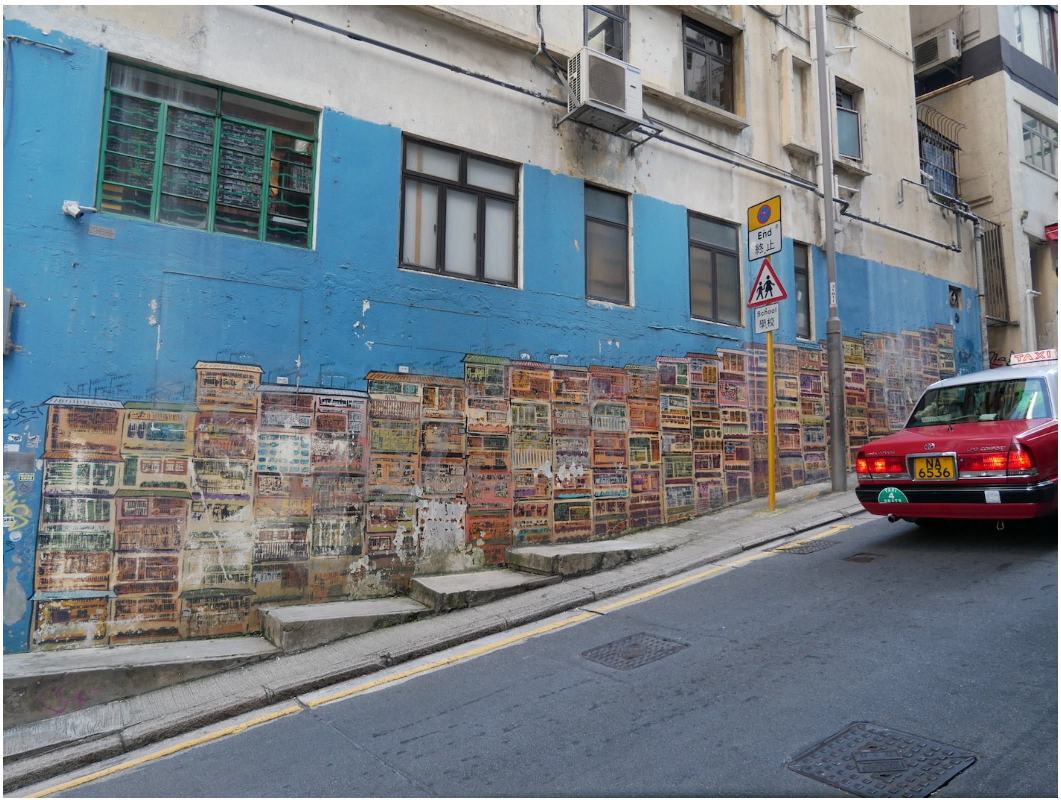

Artwork 3

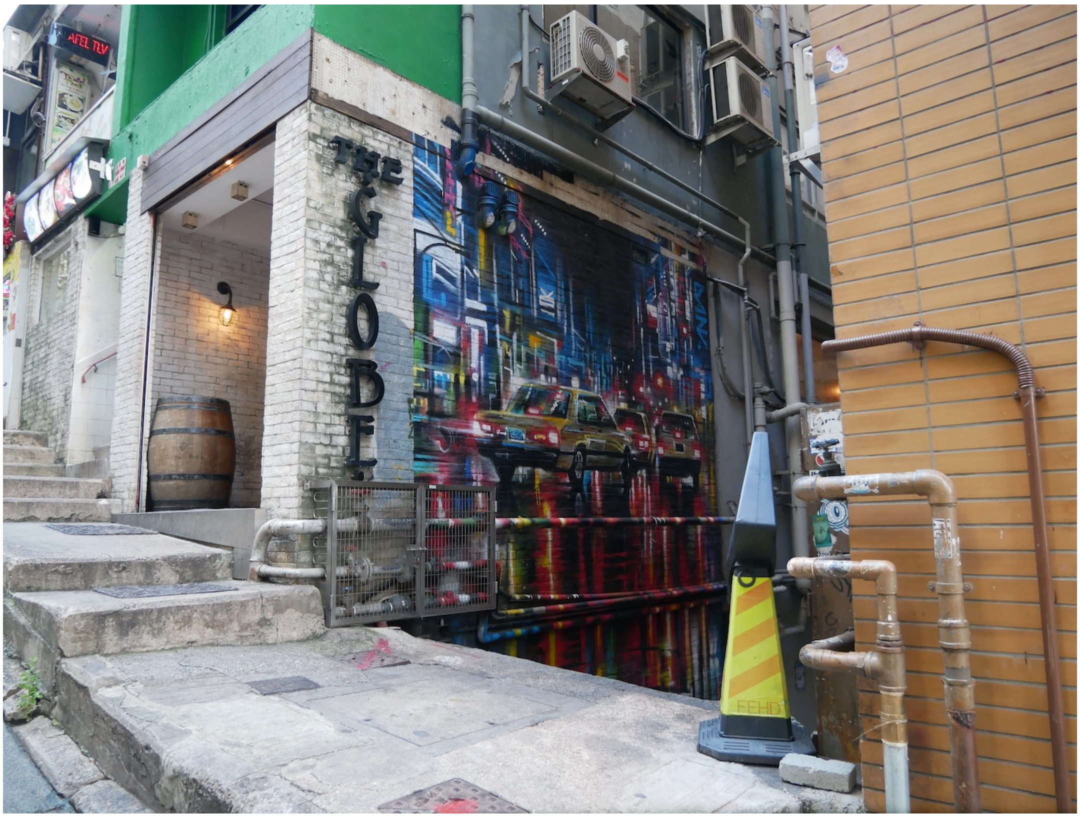


Artwork 4

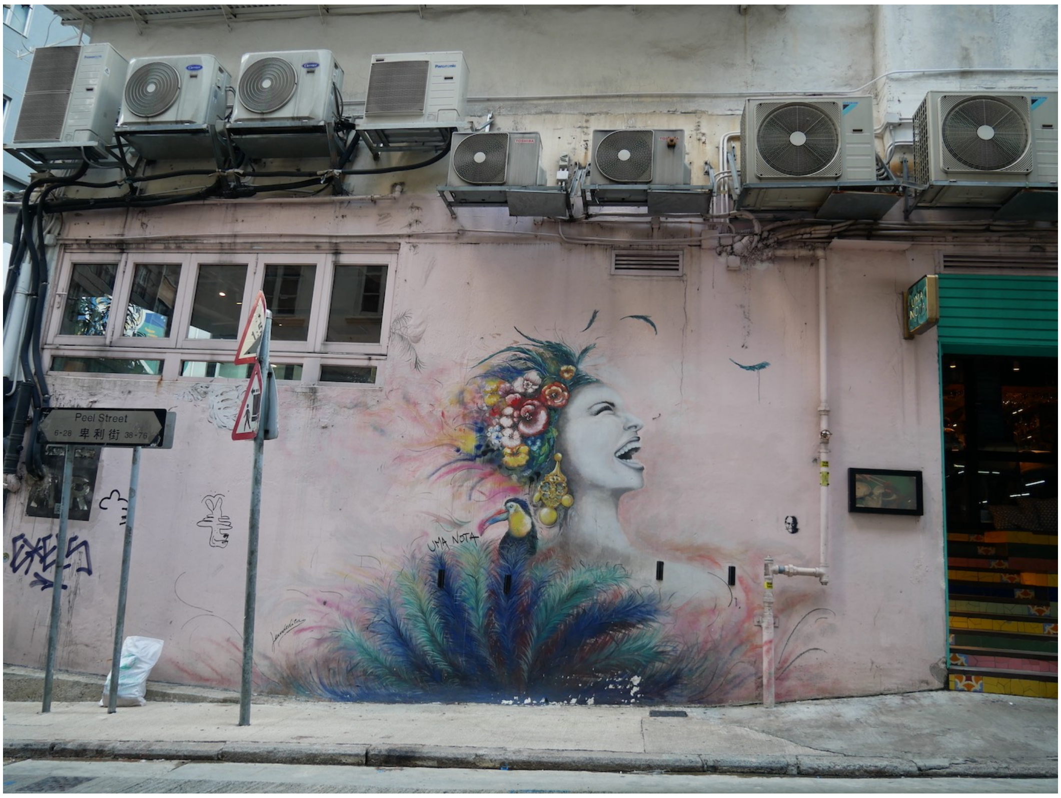

Artwork 5

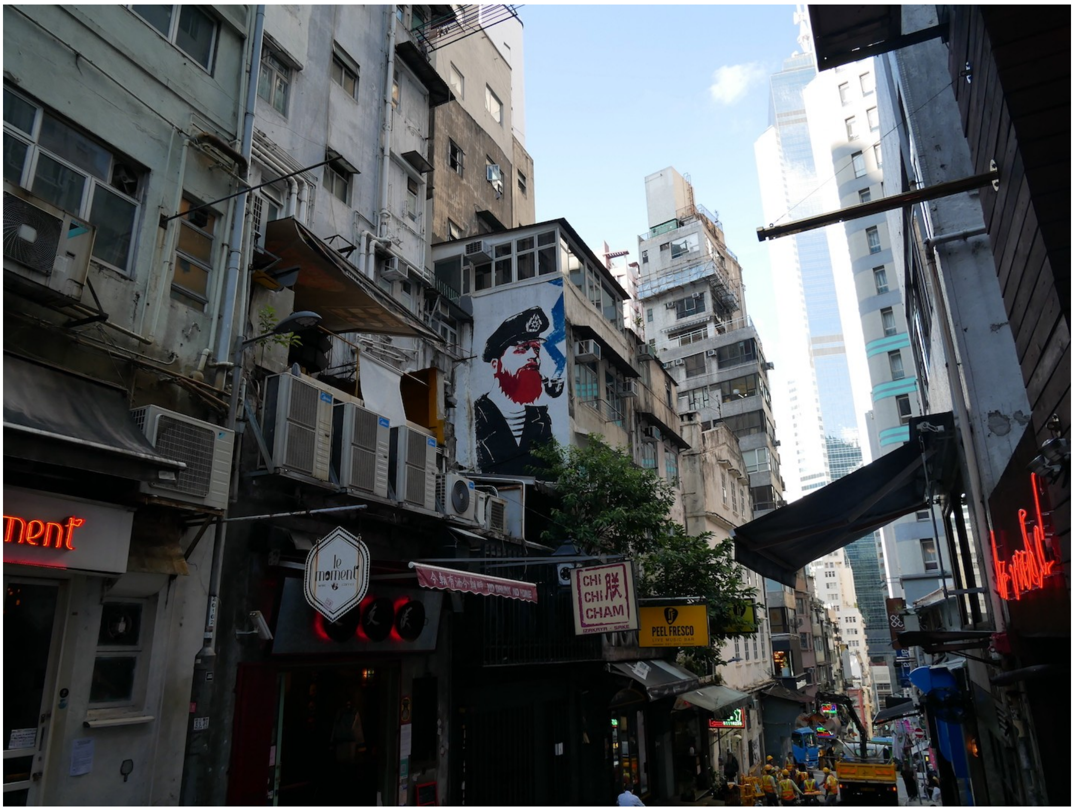


Artwork 6

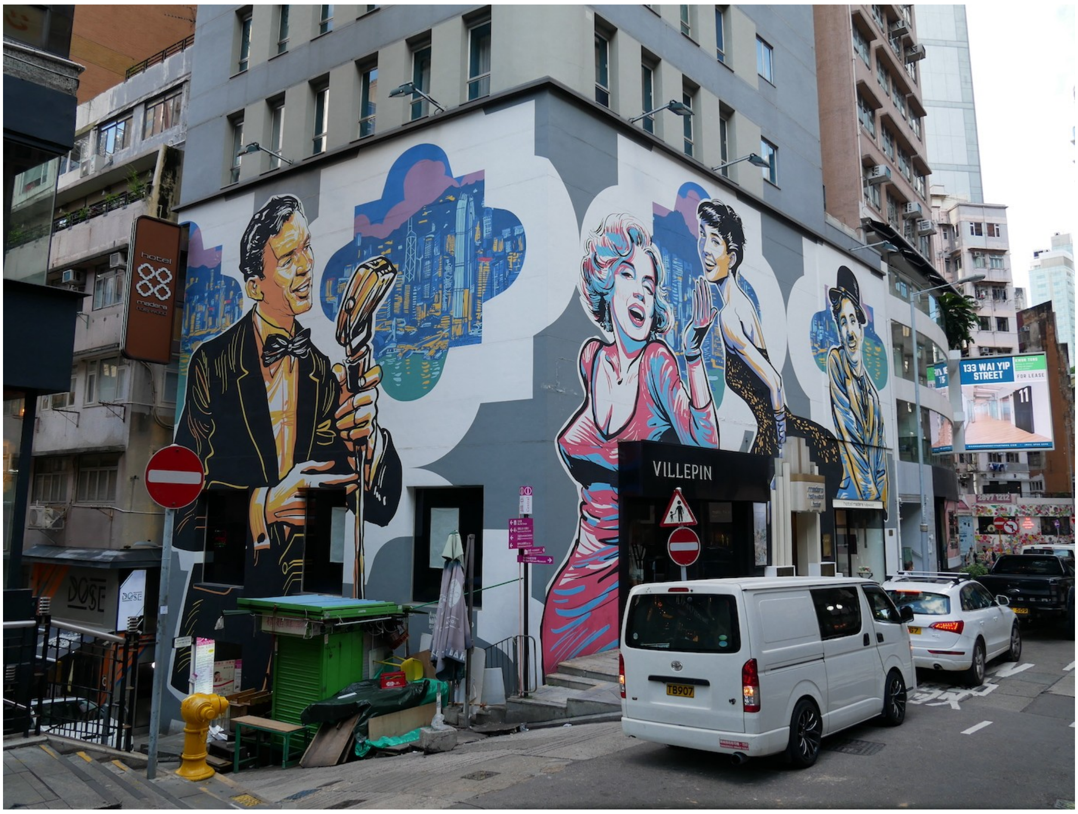

Artwork 7

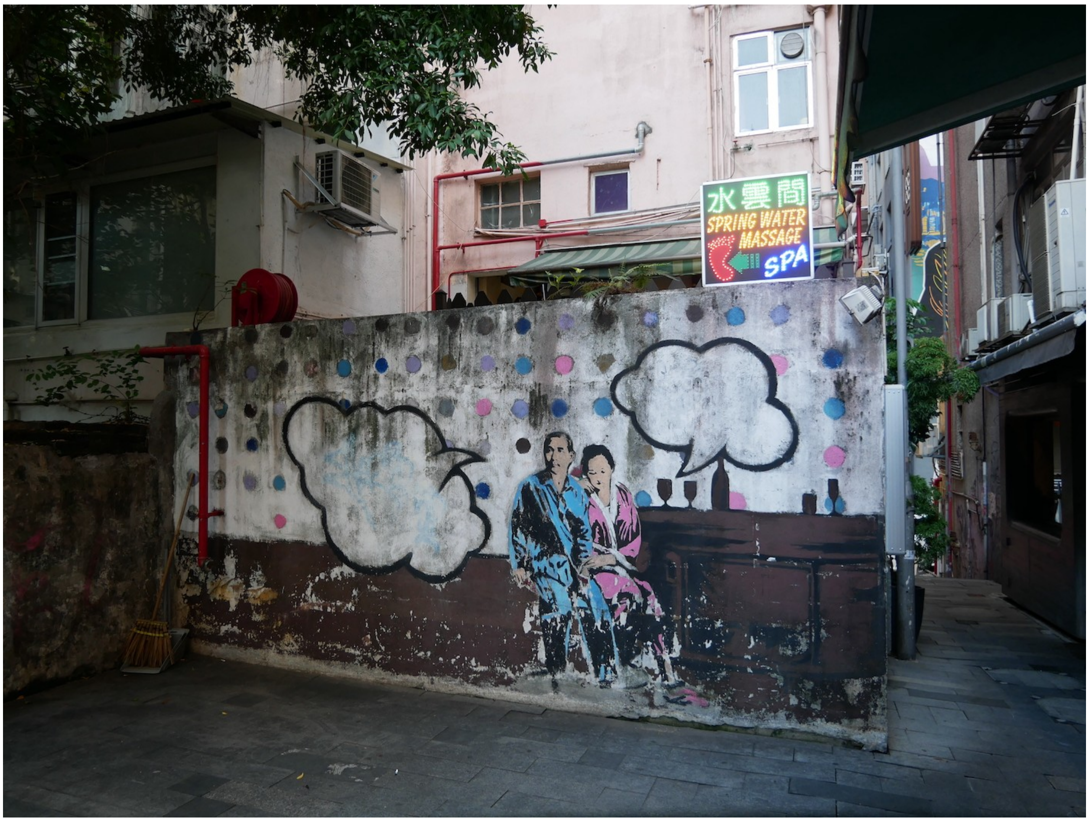


Artwork 8

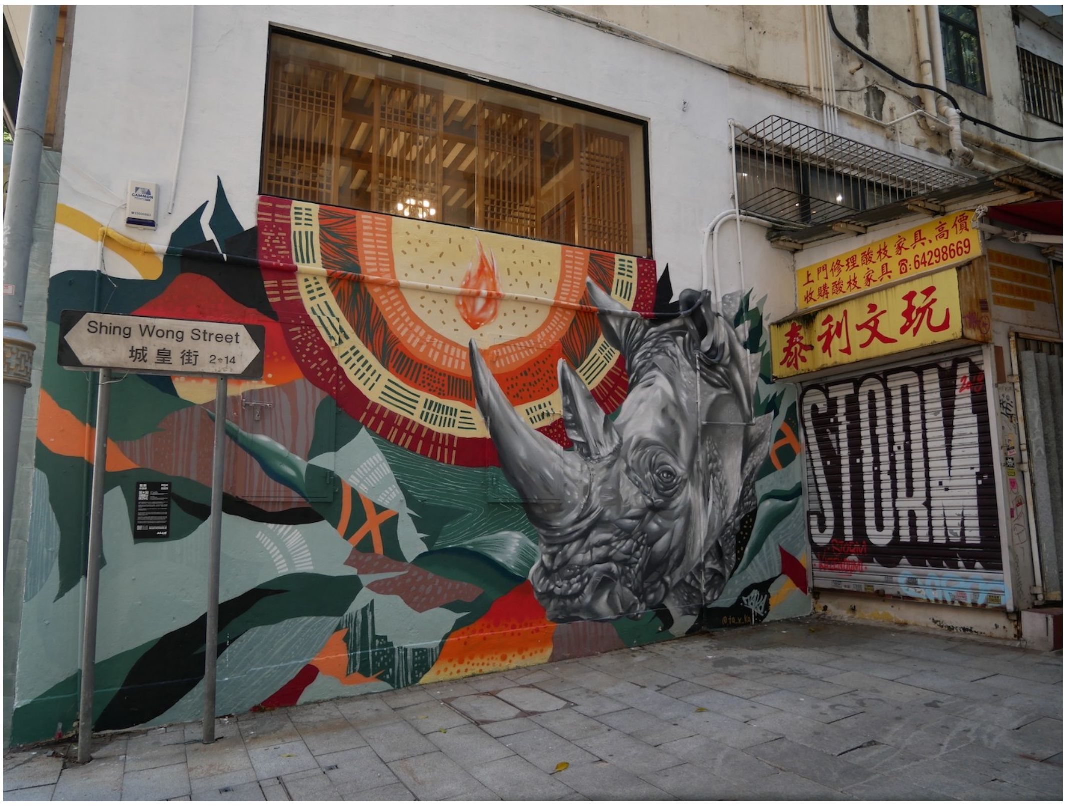

Artwork 9

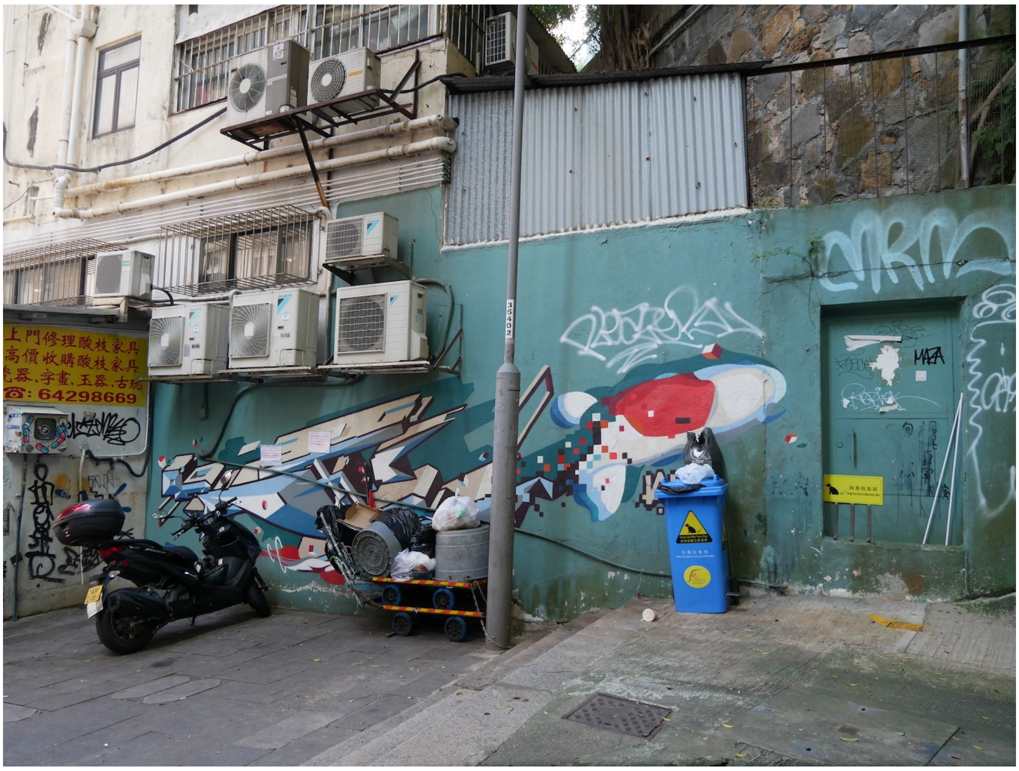


Artwork 10

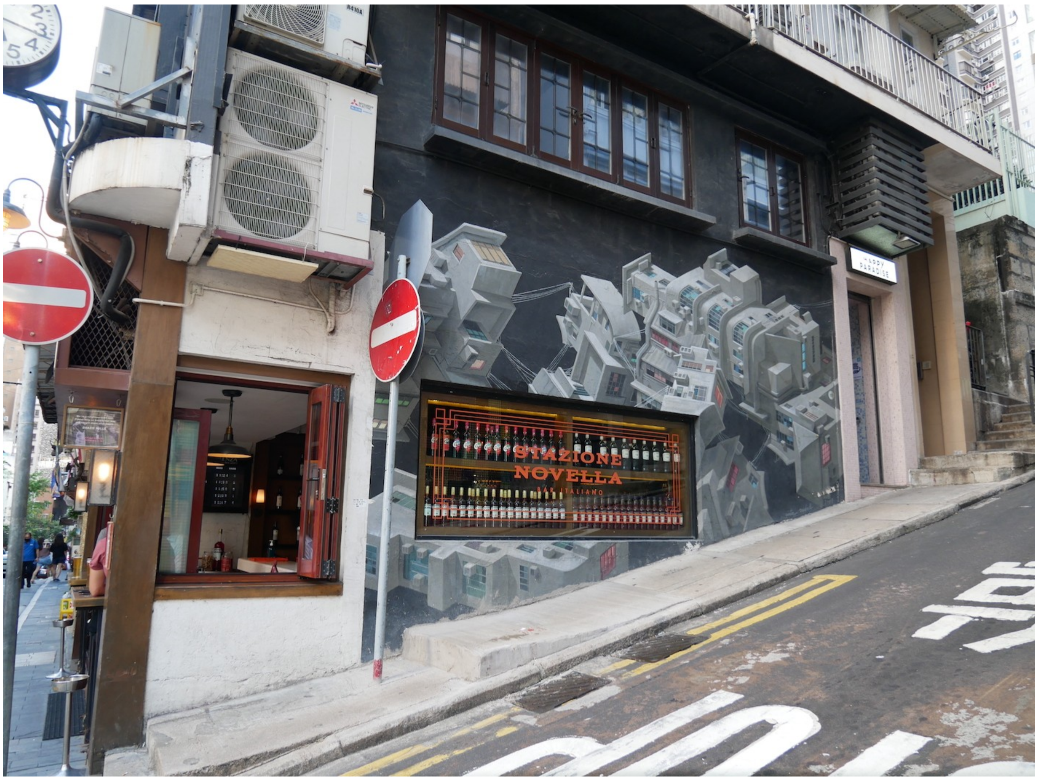

Artwork 11

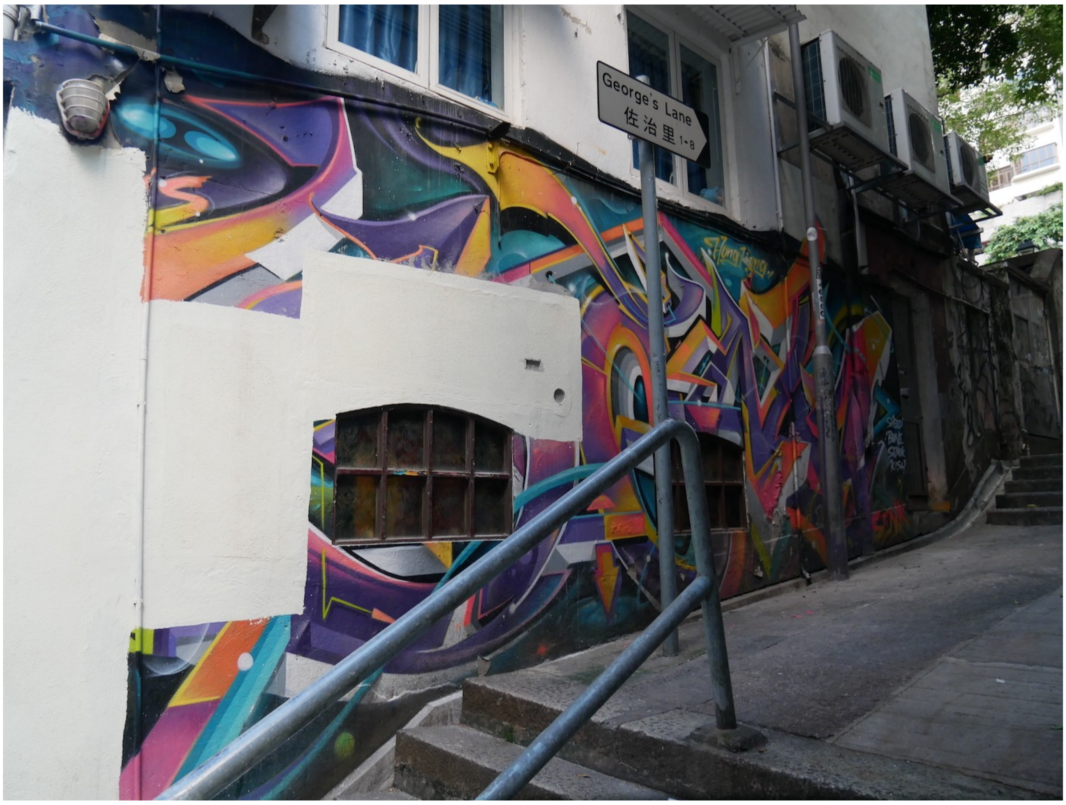


Artwork 12

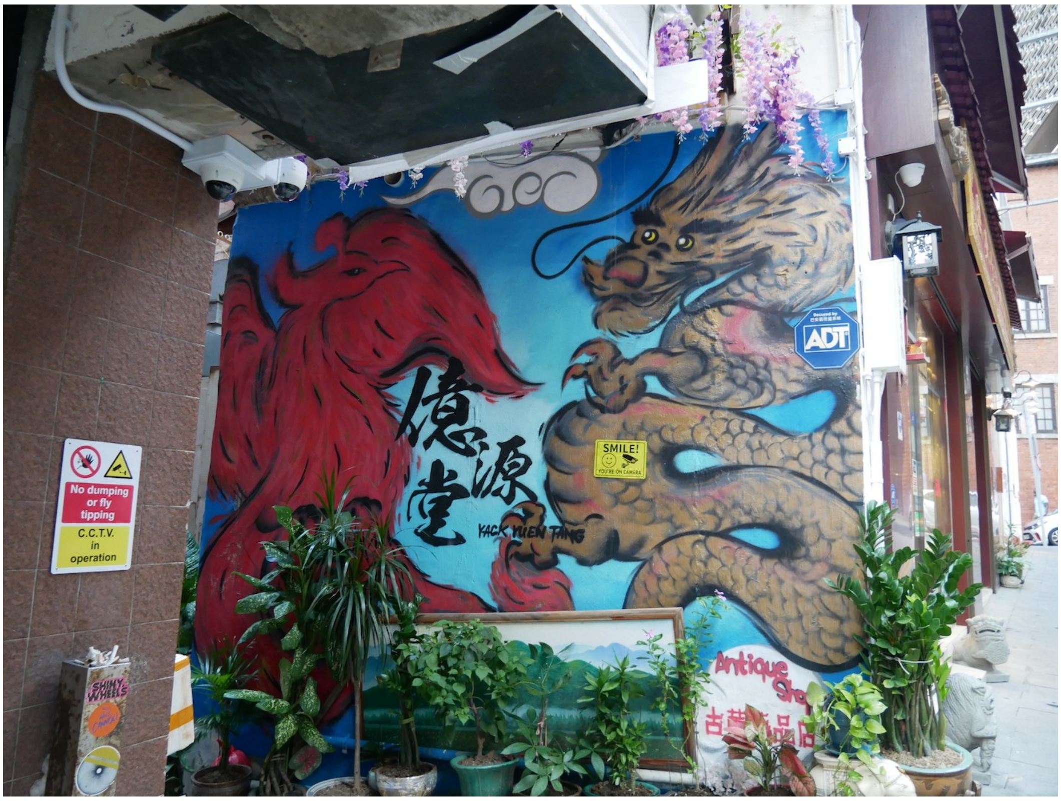

Artwork 13

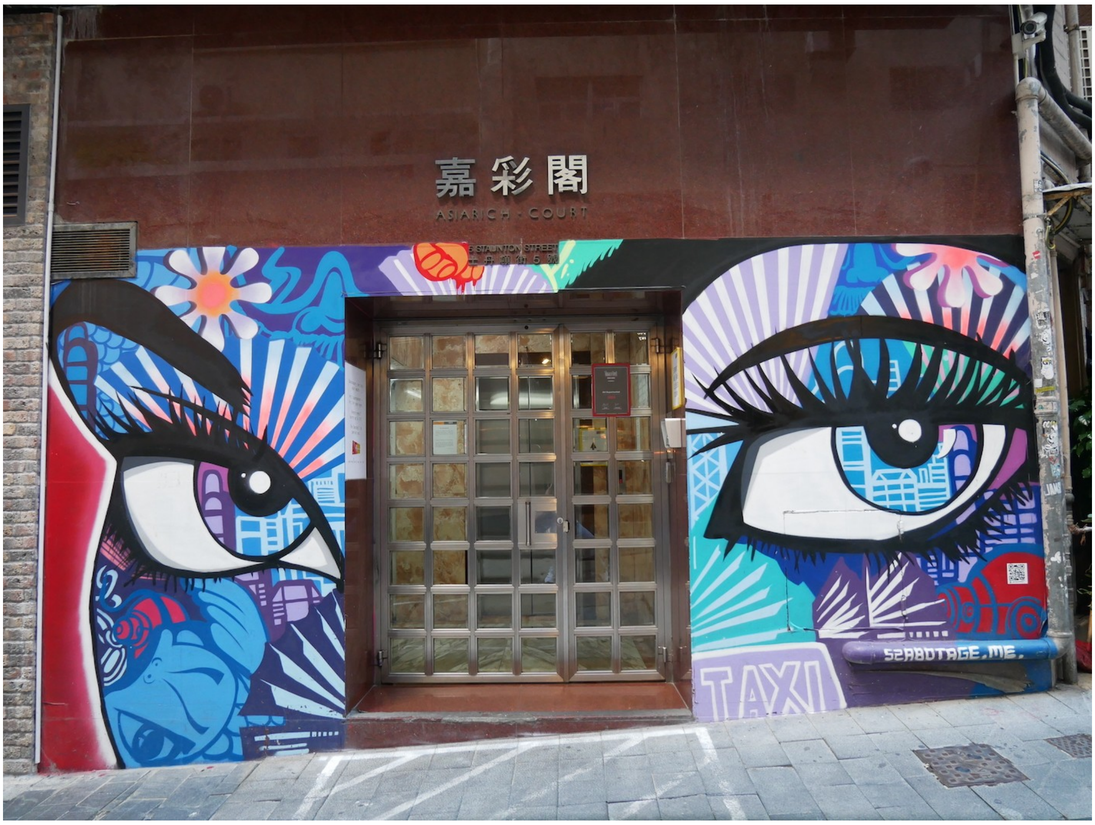


Artwork 14

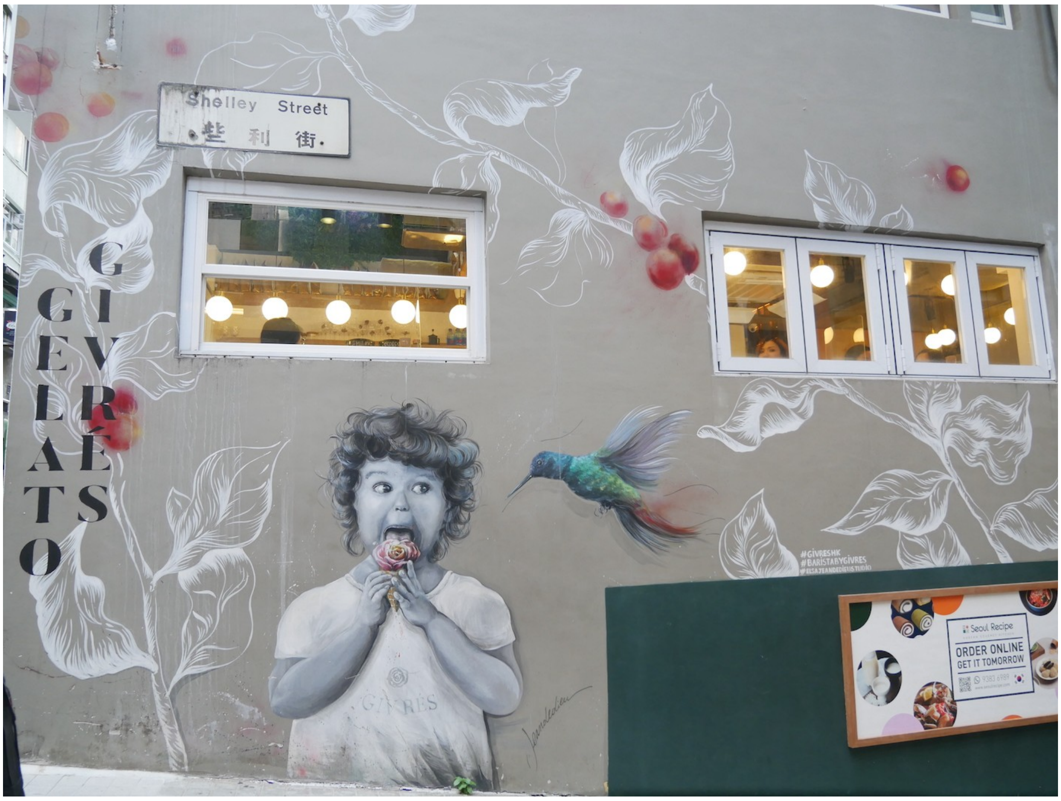

## Supplementary Material 2

## Preliminary Analyses of Experiment 1

### Need for Closure

The four experimental groups were compared on their *need for closure*. A two-way analysis of variance (ANOVA) with viewing context (street vs. laboratory) and viewing order (1–14 vs. 14–1) as between-subjects factors yielded a significant main effect of viewing context, $F(1, 96) = 5.80$, $p = .018$, $\eta_p^2 = .06$. Need for closure was higher among the street groups ($M = 56.96$, $SE = 1.39$) than the laboratory groups ($M = 52.33$, $SE = 1.33$). The main effect of viewing order was nonsignificant, $F(1, 96) = 0.22$, $p = .639$, $\eta_p^2 = .00$. The interaction between viewing context and viewing order was also nonsignificant, $F(1, 96) = 0.37$, $p = .543$, $\eta_p^2 = .00$. Descriptive statistics are presented in Table S1.

Table S1

*Need for Closure as a Function of Viewing Context and Viewing Order*

| | Street | | Laboratory | |
|---|---|---|---|---|
| | Order 1–14 | Order 14–1 | Order 1–14 | Order 14–1 |
| | *M* (*SD*) | *M* (*SD*) | *M* (*SD*) | *M* (*SD*) |
| Need for Closure | 58.00 (9.81) | 55.92 (8.82) | 52.19 (8.65) | 52.46 (10.97) |

**Expertise and Interest in Art**

The four experimental groups were compared on their self-ratings of *expertise in the visual arts*, *expertise in contemporary art*, *interest in the visual arts*, and *interest in contemporary art*. A two-way multivariate analysis of variance with viewing context (street vs. laboratory) and viewing order (1–14 vs. 14–1) as between-subjects factors did not find any significant main effect of viewing context, $F(4, 93) = 1.42$, $p = .235$, Wilks's $\Lambda = 0.94$, $\eta_p^2 = .06$, main effect of viewing order, $F(4, 93) = 0.68$, $p = .605$, Wilks's $\Lambda = 0.97$, $\eta_p^2 = .03$, nor interaction between viewing context and viewing order, $F(4, 93) = 1.20$, $p = .318$, Wilks's $\Lambda = 0.95$, $\eta_p^2 = .05$. Descriptive statistics are presented in Table S2.

Table S2

*Expertise in the Visual Arts, Expertise in Contemporary Art, Interest in the Visual Arts, and Interest in Contemporary Art as a Function of Viewing Context and Viewi*ng Order

| | Street | | Laboratory | |
|---|---|---|---|---|
| | Order 1–14 | Order 14–1 | Order 1–14 | Order 14–1 |
| | *M* (*SD*) | *M* (*SD*) | *M* (*SD*) | *M* (*SD*) |
| Expertise in the Visual Arts | 3.52 (1.41) | 3.36 (1.60) | 3.19 (1.70) | 3.04 (1.08) |
| Expertise in Contemporary Art | 3.26 (1.21) | 3.40 (1.35) | 2.88 (1.45) | 2.85 (1.41) |
| Interest in the Visual Arts | 4.83 (1.47) | 4.28 (1.82) | 4.31 (1.57) | 4.69 (1.41) |
| Interest in Contemporary Art | 4.17 (1.70) | 4.24 (1.79) | 3.50 (1.56) | 4.00 (1.77) |

**Familiarity with Artworks**

The four experimental groups were compared on their *familiarity* with the various artworks. A two-way ANOVA with viewing context (street vs. laboratory) and viewing order (1–14 vs. 14–1) as between-subjects factors yielded a significant main effect of viewing context, $F(1, 96) = 10.82$, $p = .001$, $\eta_p^2 = .10$. Familiarity was higher among participants in the street conditions ($M = 4.03$, $SE = 0.12$) than those in the laboratory conditions ($M = 3.49$, $SE = 0.12$). The main effect of viewing order was nonsignificant, $F(1, 96) = 3.14$, $p = .080$, $\eta_p^2 = .03$. The interaction between viewing context and viewing order was significant, $F(1, 96) = 7.59$, $p = .007$, $\eta_p^2 = .07$. Post hoc comparisons with Bonferroni adjustment are presented in Fig. S1.

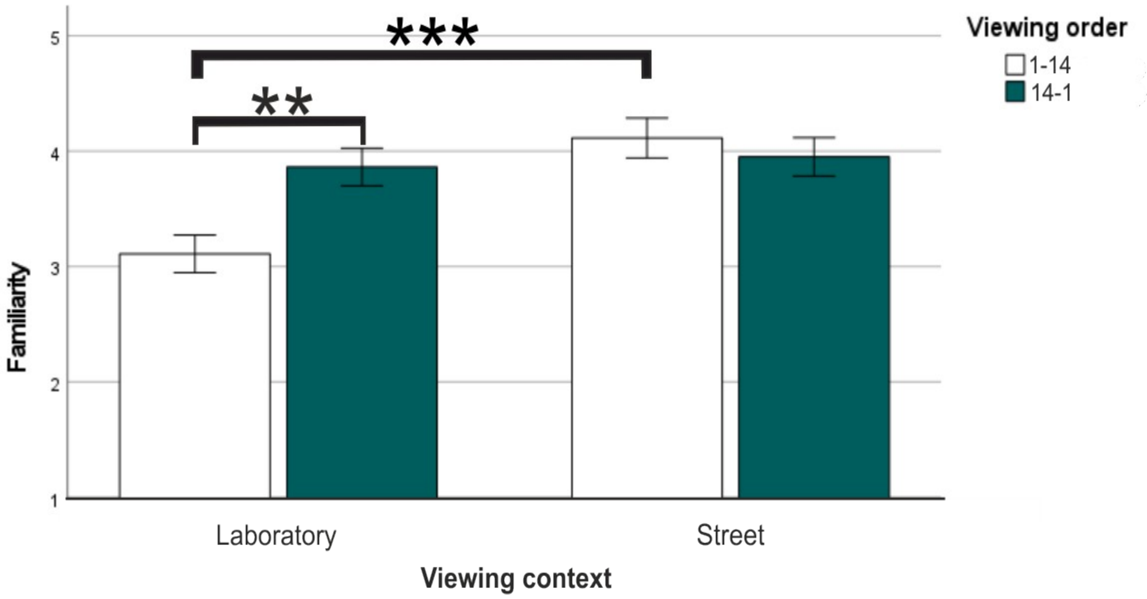


*Fig. S1*. Post hoc comparisons of the significant interaction effect of *viewing context* and *viewing order* on *familiarity with artworks*. Error bars represent ±1 standard error. ** $p < .01$, *** $p < .001$.

**Viewers' Characteristics as Predictors of Art Appreciation**

Prediction of art appreciation from viewers' personal characteristics was explored through three stepwise multiple regression analyses. Respectively, *art identification* (Table S3), *liking* (Table S4), and *understanding* (Table S5) were regressed on expertise in the visual arts, interest in the visual arts, expertise in contemporary art, interest in contemporary art, and need for closure.

Art identification could be positively predicted by interest in contemporary art alone, with an explained variance of 7%. Further, it could be predicted by interest in contemporary art (positively) combined with expertise in contemporary art (negatively), with a total explained variance of 11%.

Liking could be positively predicted by interest in contemporary art alone, with an explained variance of 17%. Further, it could be predicted by interest in contemporary art (positively) combined with expertise in contemporary art (negatively), with a total explained variance of 21%.

Understanding could be positively predicted by interest in contemporary art alone, with an explained variance of 20%. There were no further significant models.

Table S3

*Multiple Regression of Art Identification*

| | Model 1 | | Model 2 | |
|---|---|---|---|---|
| | $F(1, 98) = 7.91$, $p = .006$, $R^2_{adj} = .065$ | | $F(2, 97) = 6.98$, $p = .001$, $R^2_{adj} = .108$ | |
| | β | *p* | β | *p* |
| *Predictors* | | | | |
| Interest in Contemporary Art | .27 | .006 | .45 | < .001 |
| Expertise in Contemporary Art | - | - | –.29 | .019 |
| *Variables Excluded* | | | | |
| Need for Closure | .06 | .535 | .04 | .693 |
| Expertise in the Visual Arts | –.08 | .491 | .13 | .345 |
| Interest in the Visual Arts | –.01 | .960 | .10 | .481 |
| Expertise in Contemporary Art | –.29 | .019 | - | - |

Table S4

*Multiple Regression of Liking*

| | Model 1 | | Model 2 | |
|---|---|---|---|---|
| | $F(1, 98) = 21.29$, $p < .001$, $R^2_{adj} = .170$ | | $F(2, 97) = 14.05$, $p < .001$, $R^2_{adj} = .209$ | |
| | β | *p* | β | *p* |
| *Predictors* | | | | |
| Interest in Contemporary Art | .42 | < .001 | .59 | < .001 |
| Expertise in Contemporary Art | - | - | –.27 | .018 |
| *Variables Excluded* | | | | |
| Need for Closure | .11 | .231 | .09 | .324 |
| Expertise in the Visual Arts | –.18 | .095 | –.04 | .767 |
| Interest in the Visual Arts | –.19 | .147 | –.11 | .430 |
| Expertise in Contemporary Art | –.27 | .018 | - | - |

Table S5

*Multiple Regression of Understanding*

| | Model 1 | |
|---|---|---|
| | $F(1, 98) = 26.41$, $p < .001$, $R^2_{adj} = .204$ | |
| | β | *p* |
| *Predictors* | | |
| Interest in Contemporary Art | .46 | < .001 |
| *Variables Excluded* | | |
| Need for Closure | .12 | .196 |
| Expertise in the Visual Arts | .15 | .135 |
| Interest in the Visual Arts | –.04 | .783 |
| Expertise in Contemporary Art | .06 | .585 |

## Supplementary Material 3

## Digital Reproductions of Artworks Used in Experiment 2

The numbering of the artworks corresponds to the order in which they were shown to the participants.

Artwork 1

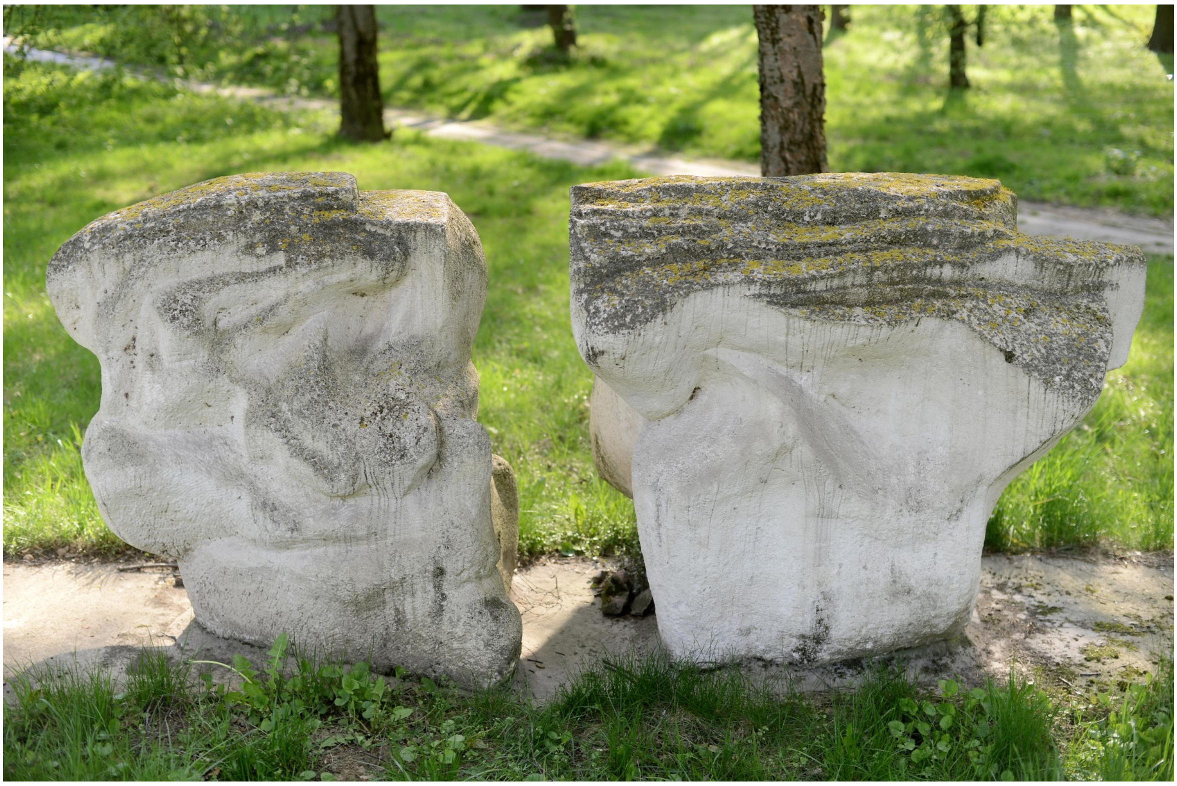

Artwork 2

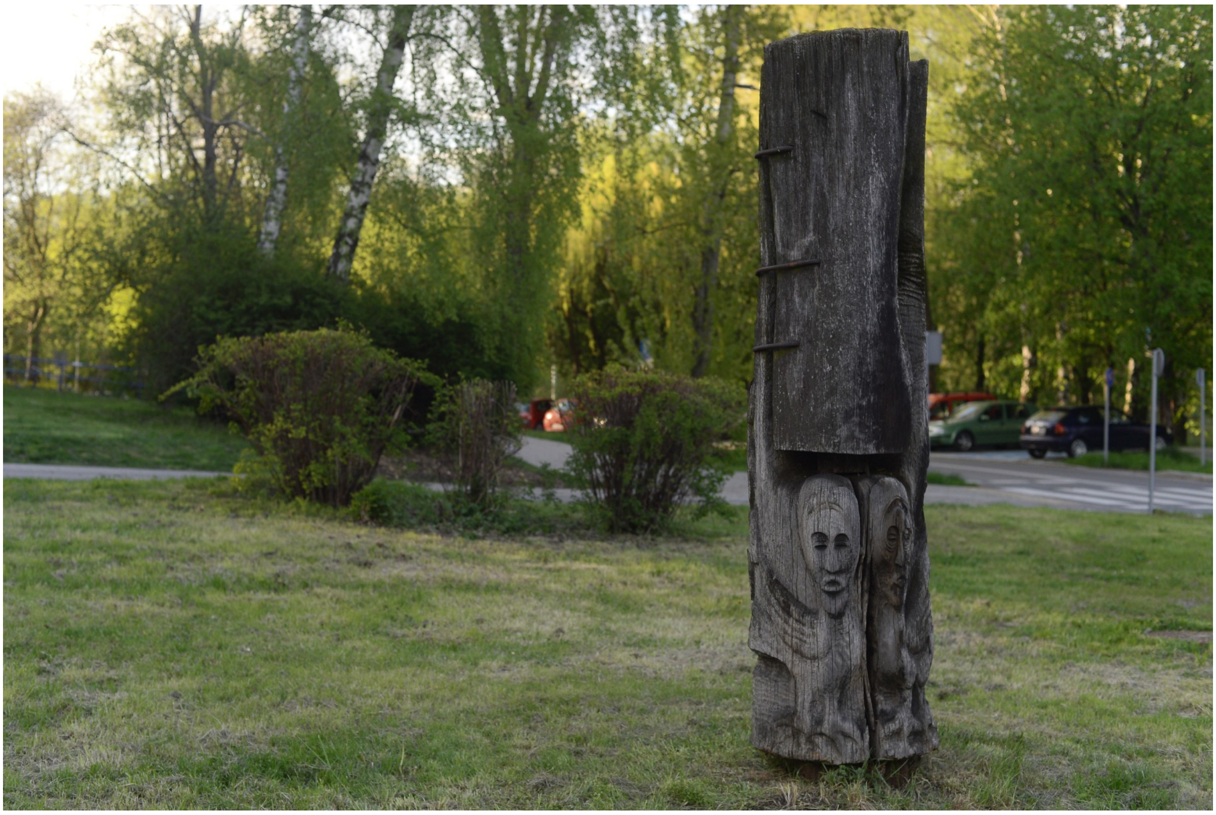

Artwork 3

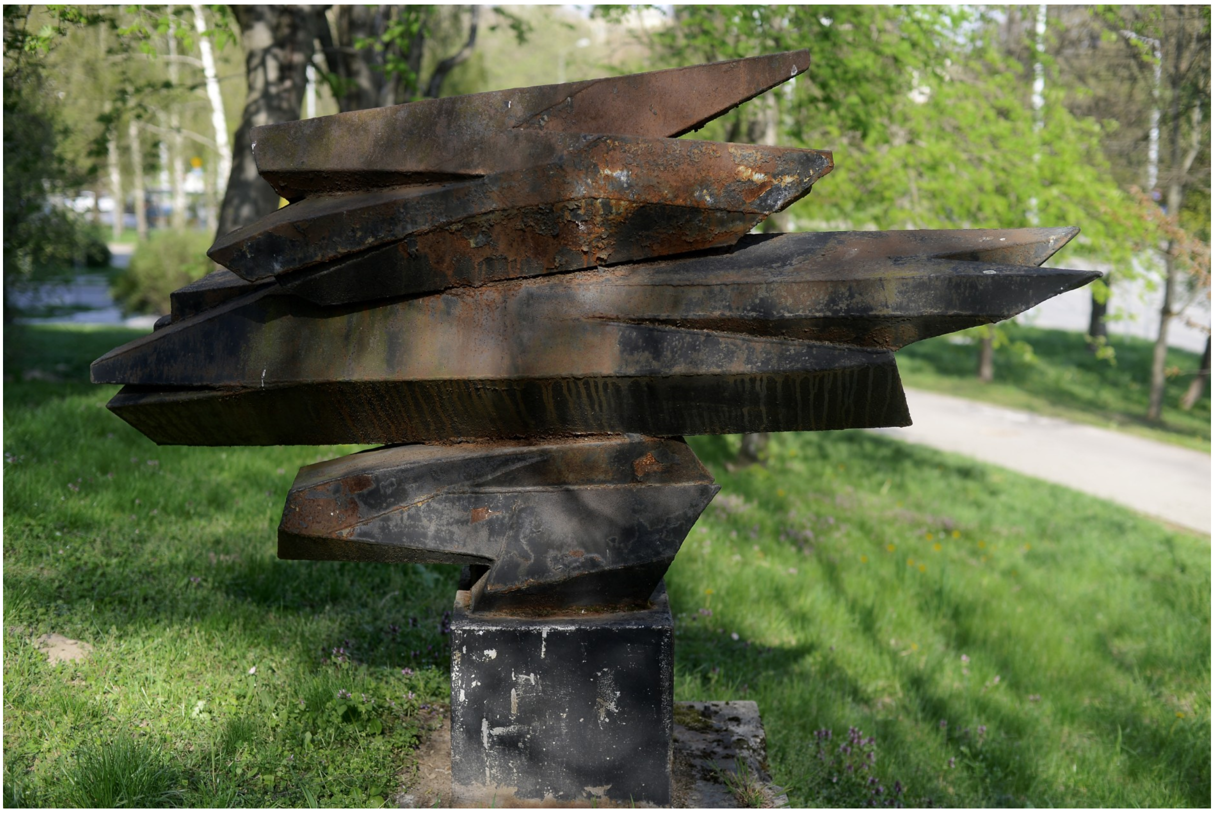

Artwork 4

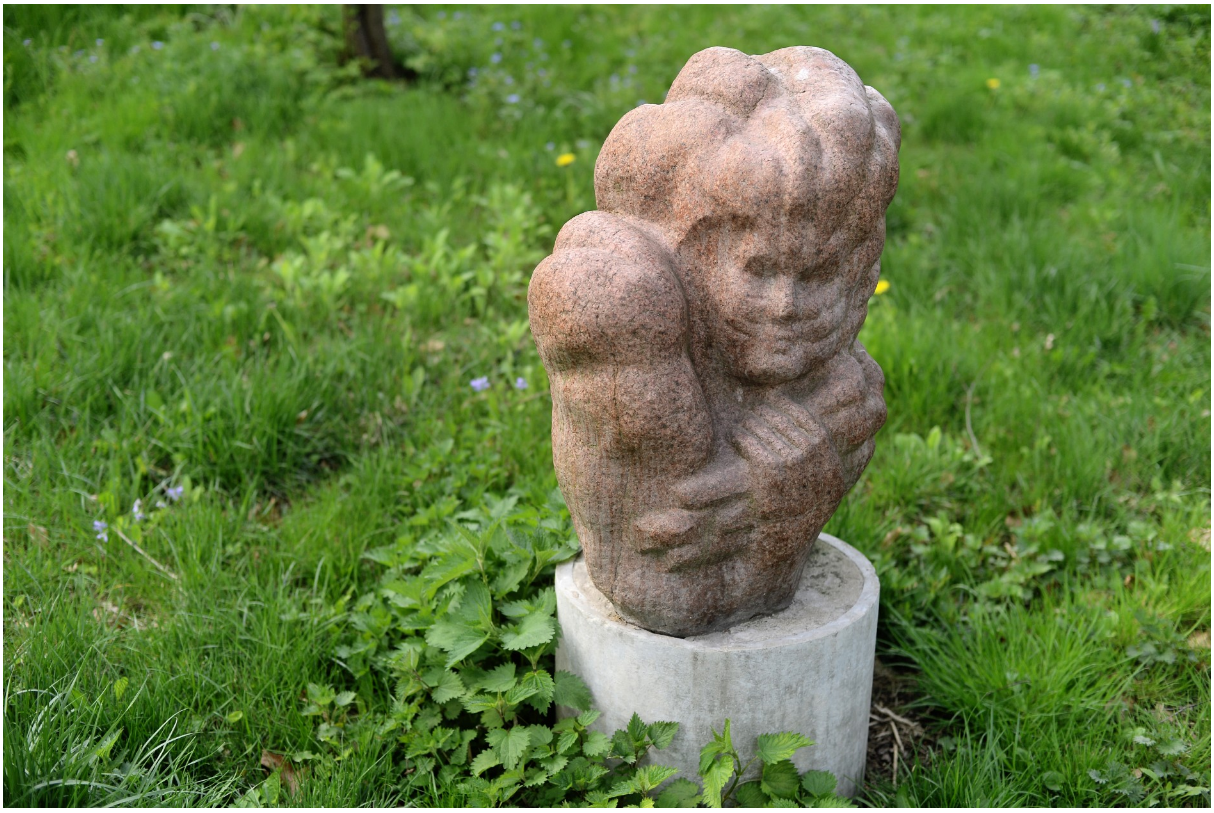

Artwork 5

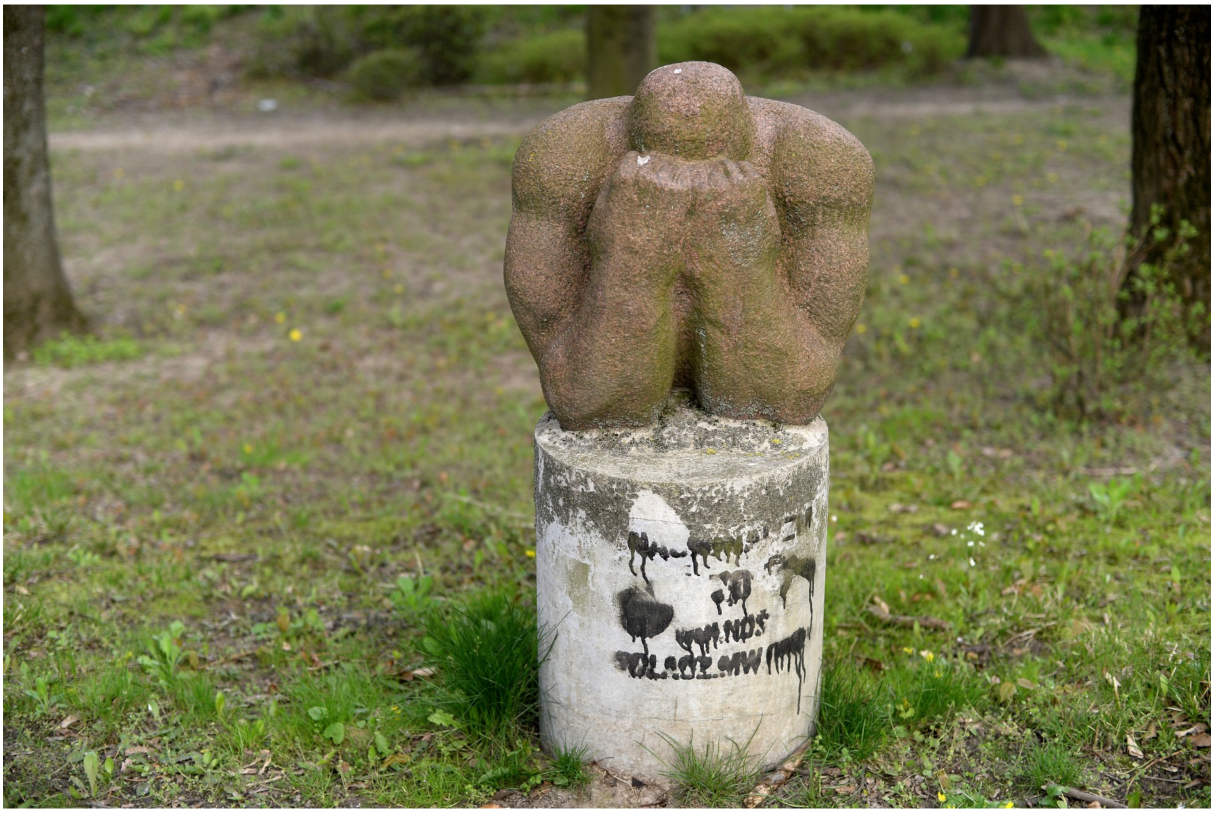

Artwork 6

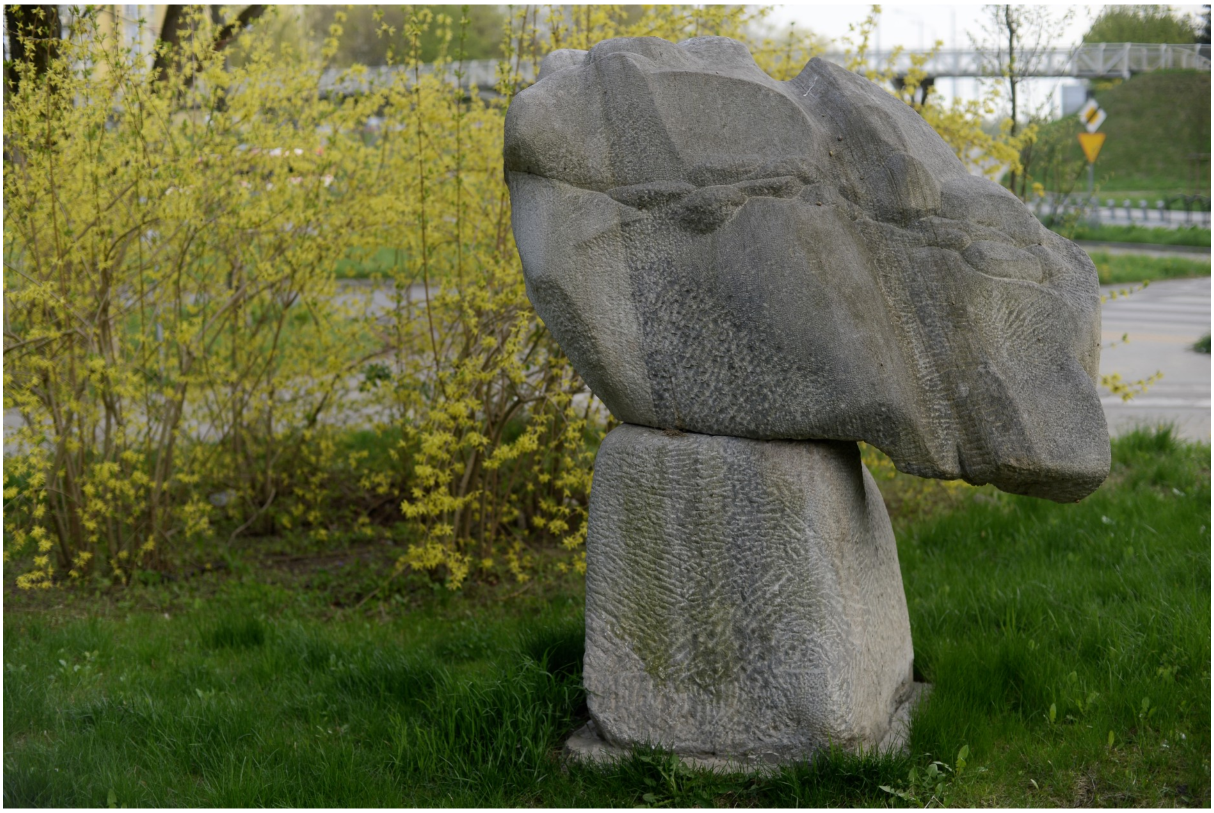

Artwork 7

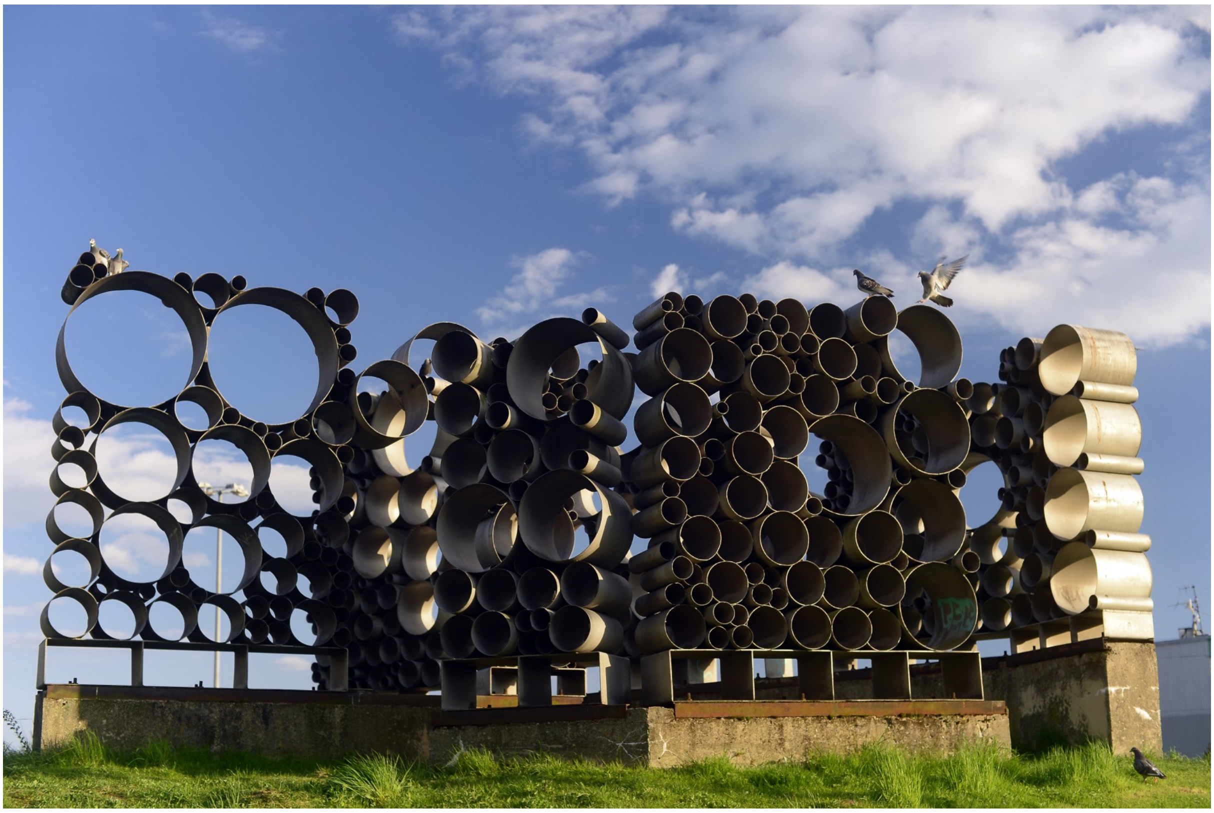

Artwork 8

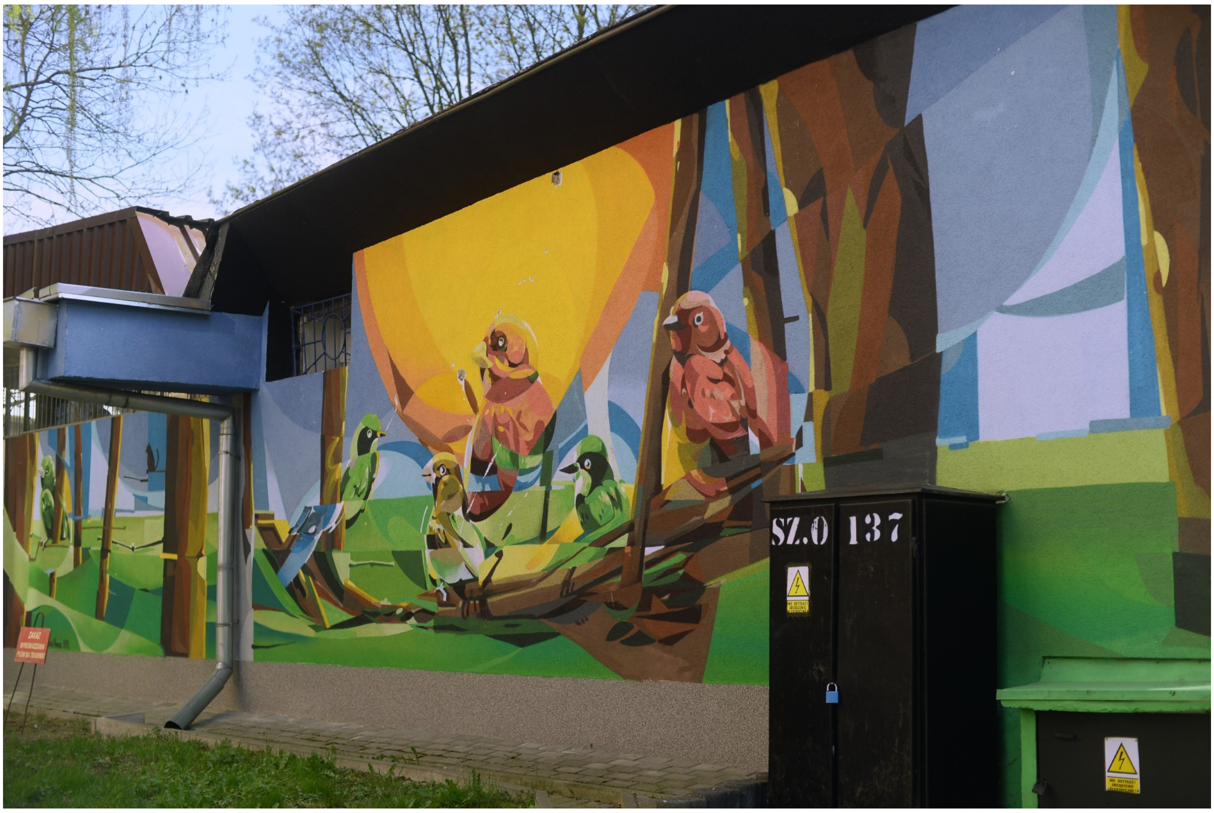

Artwork 9

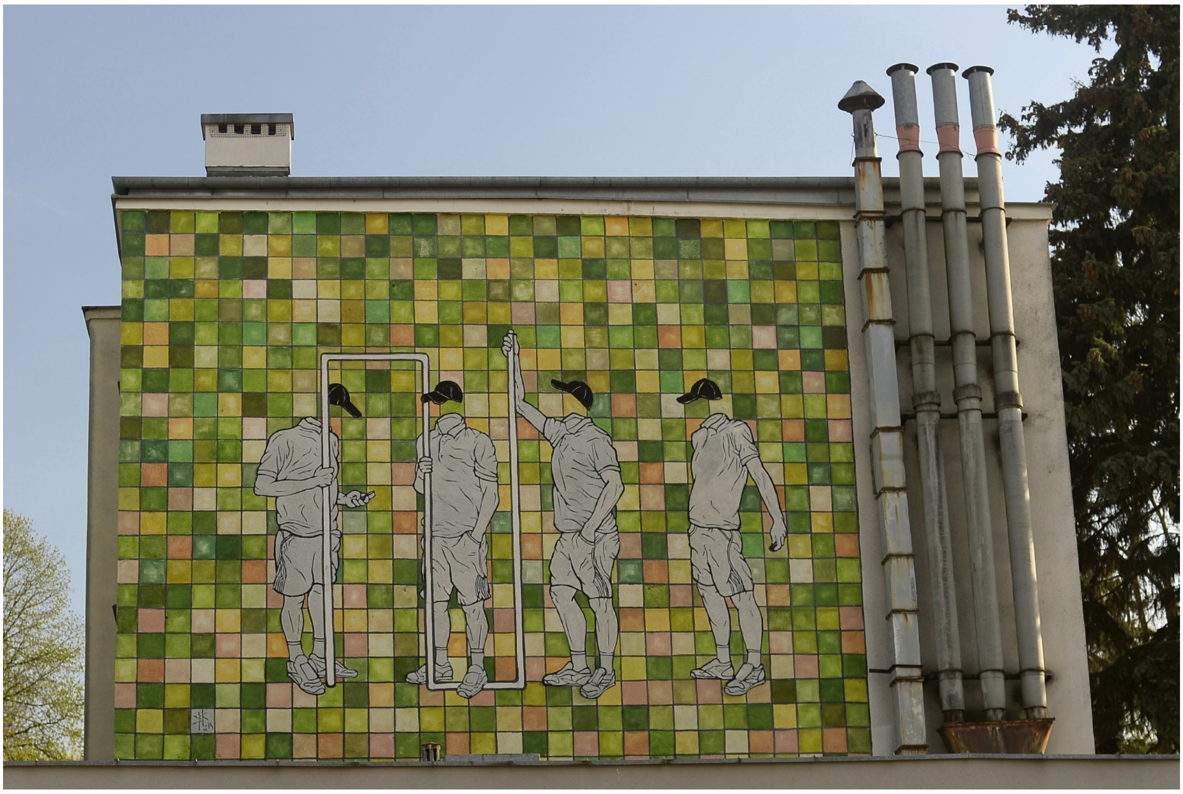

Artwork 10

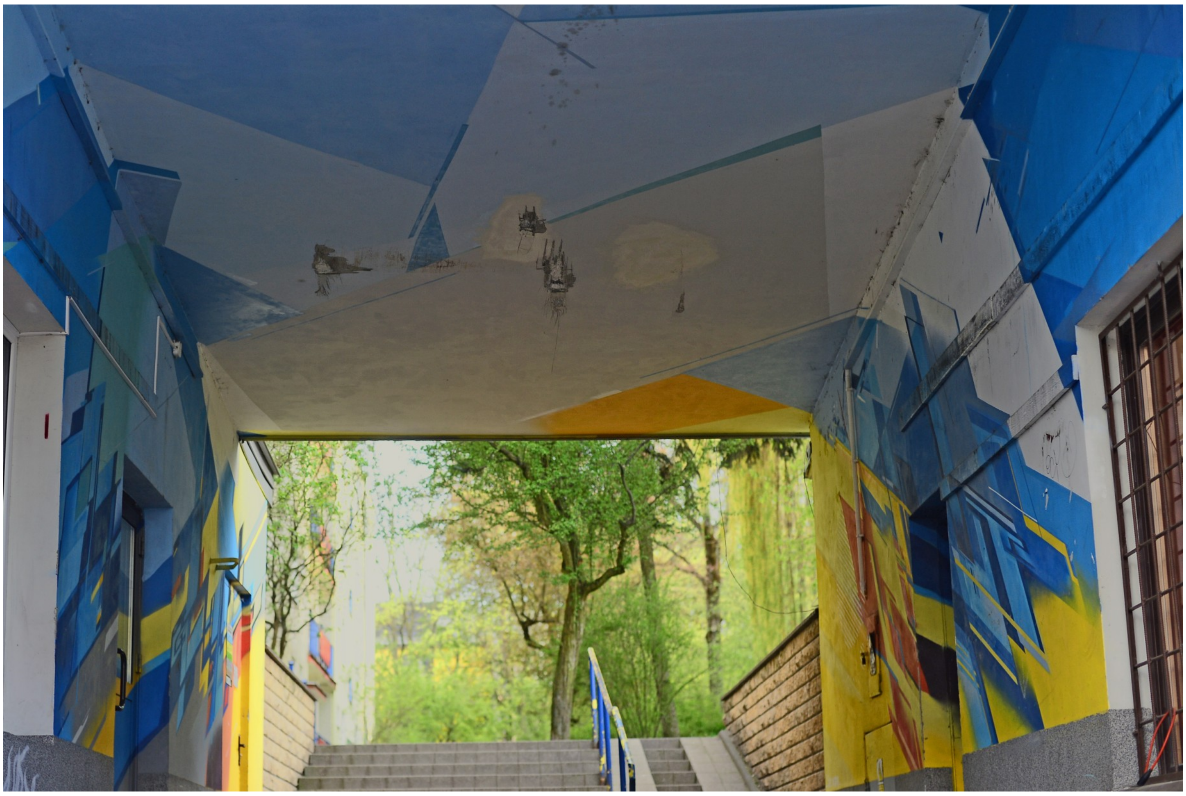

Artwork 11

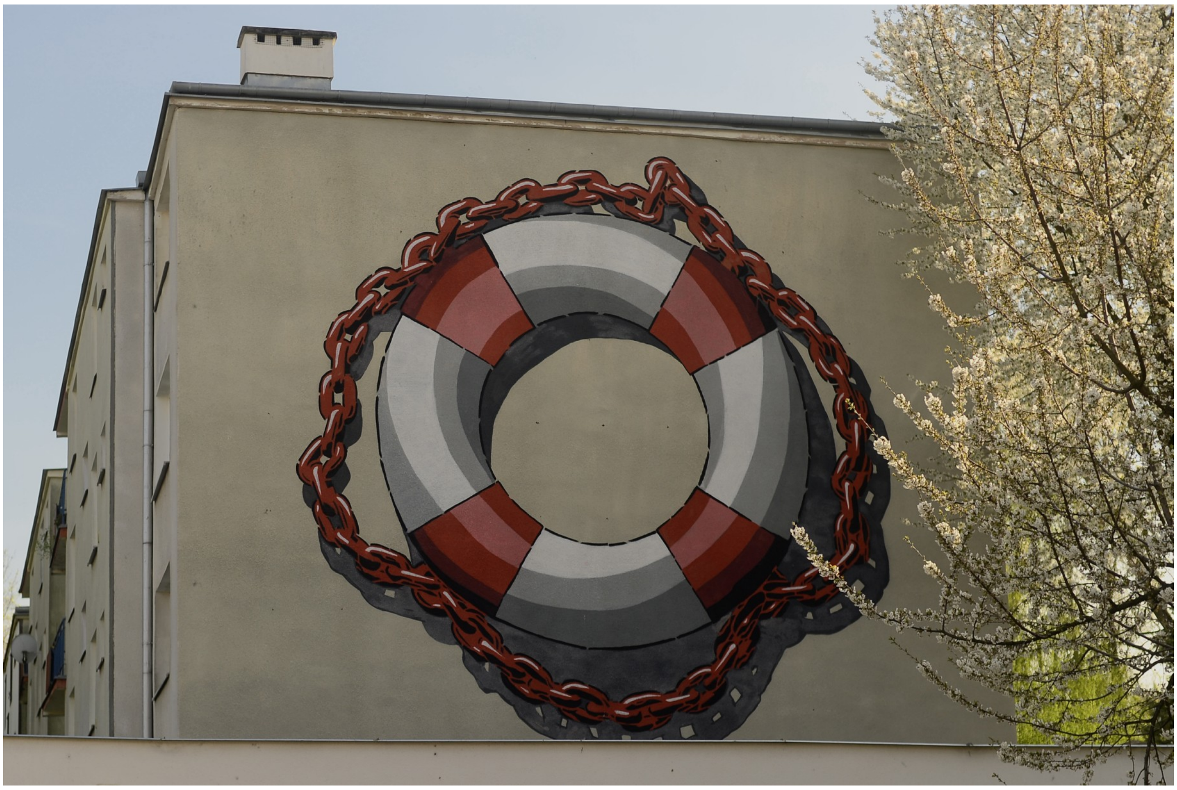

Artwork 12

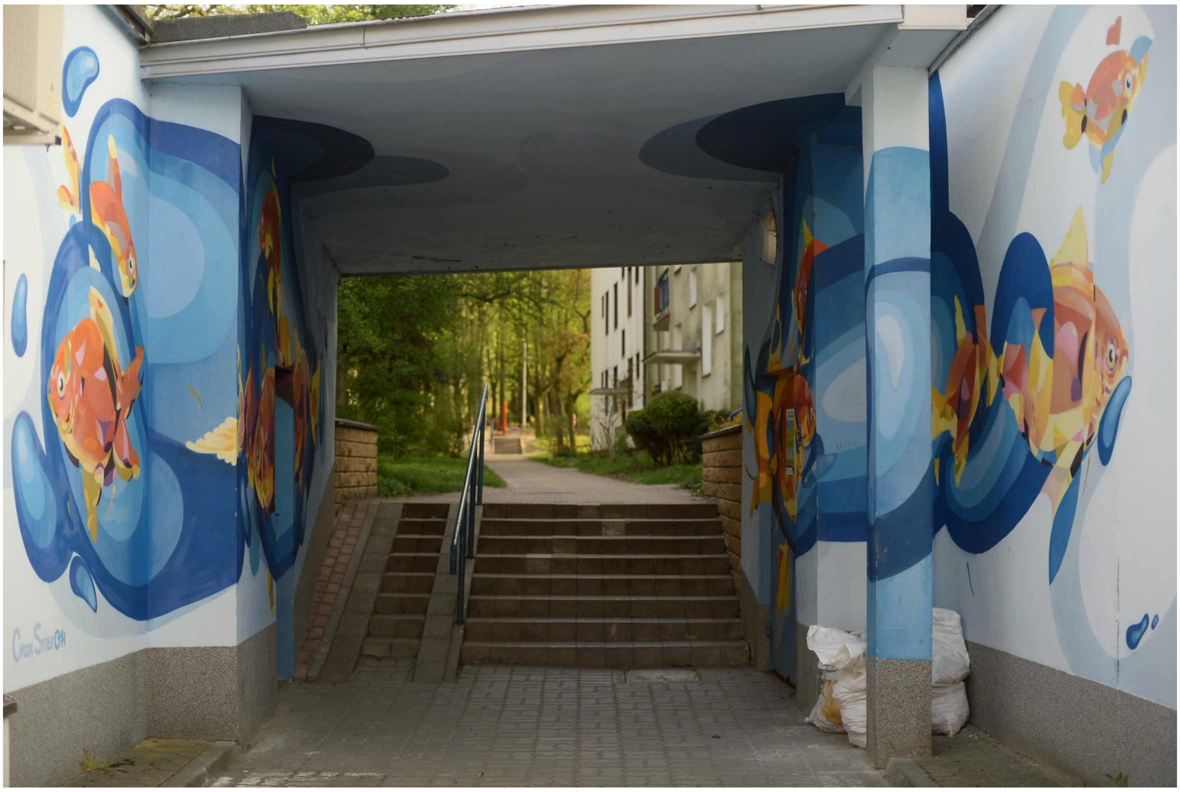

Artwork 13

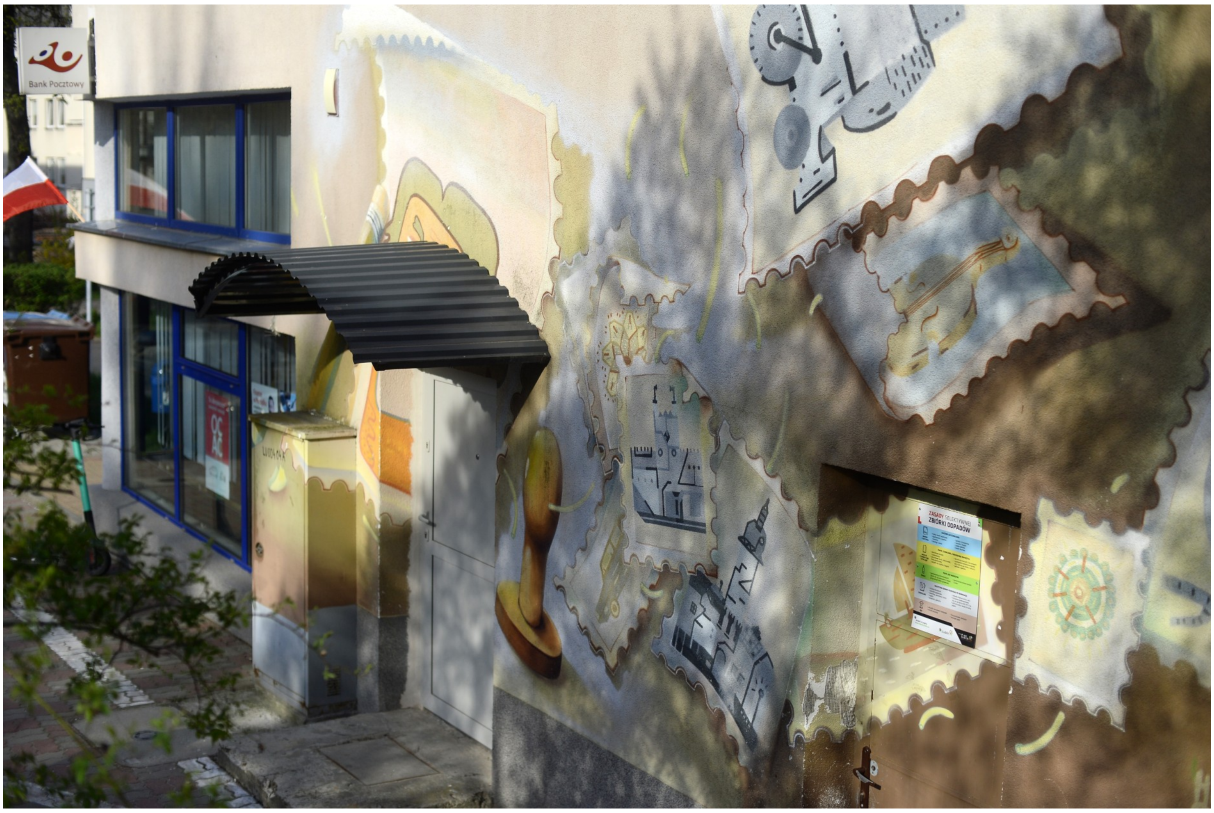


Artwork 14

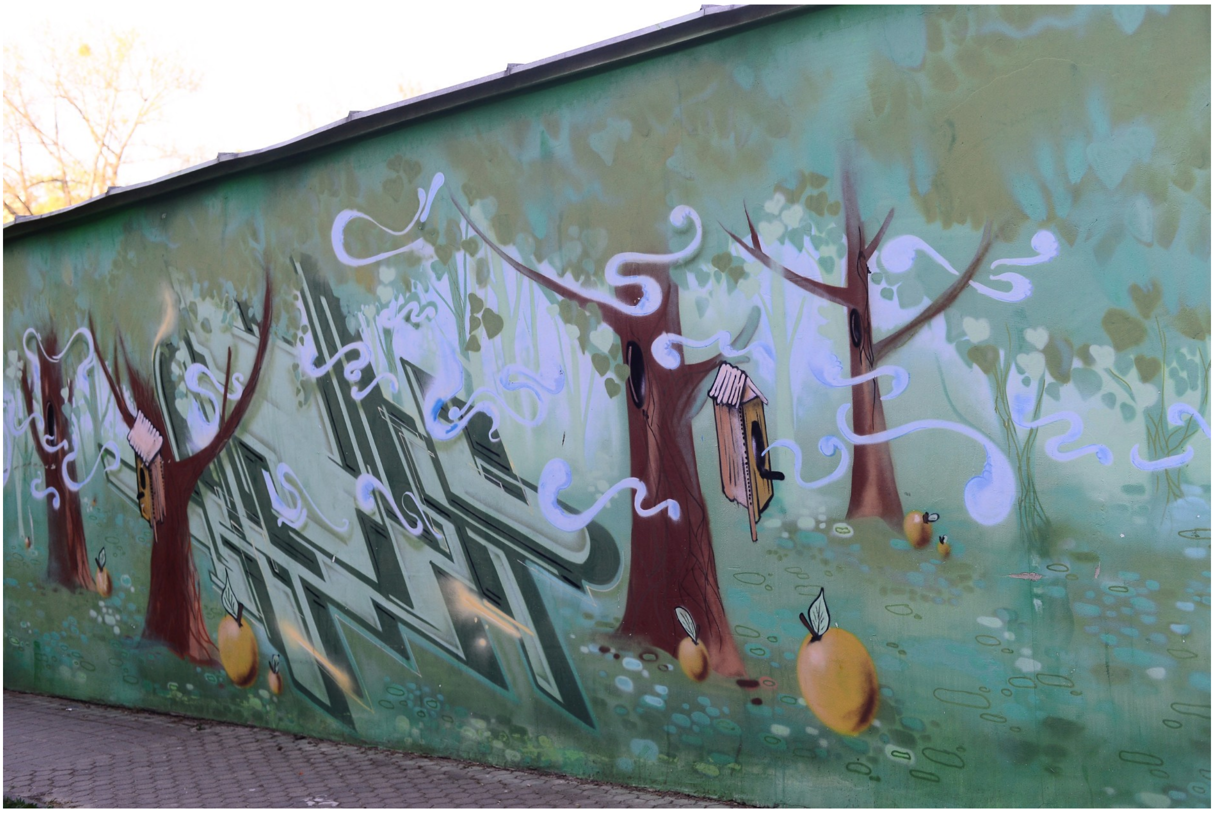

**Supplementary Material 4**

**Preliminary Analyses of Experiment 2**

## Need for Closure

The four experimental groups were compared on their *need for closure*. A two-way analysis of variance (ANOVA) with viewing context (street vs. laboratory) and viewing order (1–14 vs. 14–1) as between-subjects factors yielded a significant main effect of viewing context, $F(1, 82) = 4.74$, $p = .032$, $\eta_p^2 = .06$. The main effect of viewing order was nonsignificant, $F(1, 82) = 0.11$, $p = .745$, $\eta_p^2 = .00$. The interaction between viewing context and viewing order was significant, $F(1, 82) = 7.02$, $p = .010$, $\eta_p^2 = .08$. Post hoc comparisons with Bonferroni adjustment are presented in Fig. S2.

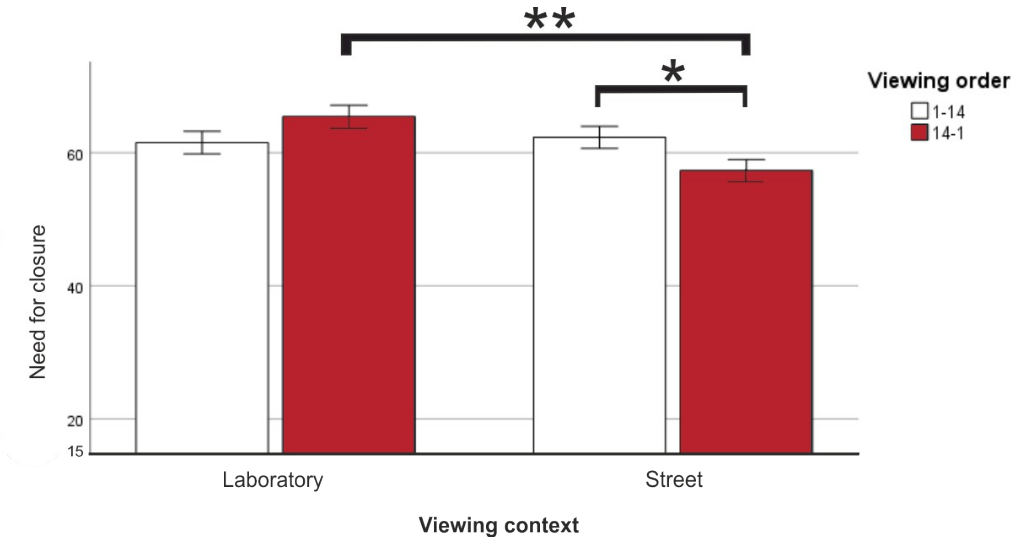


*Fig. S2*. Post hoc comparisons of the significant interaction effect of *viewing context* and *viewing order* on *need for closure*. Error bars represent ±1 standard error. * $p < .05$, ** $p < .01$.

## Expertise and Interest in Art

The four experimental groups were compared on their self-ratings of *expertise in the visual arts* and *interest in contemporary art*. A two-way multivariate analysis of variance

with viewing context (street vs. laboratory) and viewing order (1–14 vs. 14–1) as between-subjects factors did not find any significant main effect of viewing context, $F(2, 83) = 1.51$, $p = .228$, Wilks's $\Lambda = 0.97$, $\eta_p^2 = .04$, main effect of viewing order, $F(2, 83) = 0.53$, $p = .593$, Wilks's $\Lambda = 0.99$, $\eta_p^2 = .01$, nor interaction between viewing context and viewing order, $F(2, 83) = 1.04$, $p = .359$, Wilks's $\Lambda = 0.98$, $\eta_p^2 = .02$. Descriptive statistics are presented in Table S6.

Table S6

*Expertise in the Visual Arts and Interest in Contemporary Art as a Function of Viewing Context and Viewing O*rder

| | Street | | Laboratory | |
|---|---|---|---|---|
| | Order 1–14 | Order 14–1 | Order 1–14 | Order 14–1 |
| | *M* (*SD*) | *M* (*SD*) | *M* (*SD*) | *M* (*SD*) |
| Expertise in the Visual Arts | 2.86 (1.17) | 2.59 (1.10) | 2.32 (1.17) | 2.23 (1.54) |
| Interest in Contemporary Art | 2.91 (1.54) | 3.32 (1.29) | 3.05 (1.46) | 2.82 (1.65) |

**Familiarity with Artworks**

The four experimental groups were compared on their familiarity with the various artworks. A two-way ANOVA with viewing context (street vs. laboratory) and viewing order (1–14 vs. 14–1) as between-subjects factors yielded a significant main effect of viewing context, $F(1, 84) = 6.49$, $p = .013$, $\eta_p^2 = .07$. Familiarity was higher among participants in the street conditions ($M = 3.75$, $SE = 0.18$) than those in the laboratory conditions ($M = 3.12$, $SE = 0.18$). The main effect of viewing order was nonsignificant, $F(1, 84) = 0.01$, $p = .923$, $\eta_p^2 = .00$. The interaction between viewing context and viewing order was significant, $F(1, 84) = 8.18$, $p = .005$, $\eta_p^2 = .09$. Post hoc comparisons with Bonferroni adjustment are presented in Fig. S3.

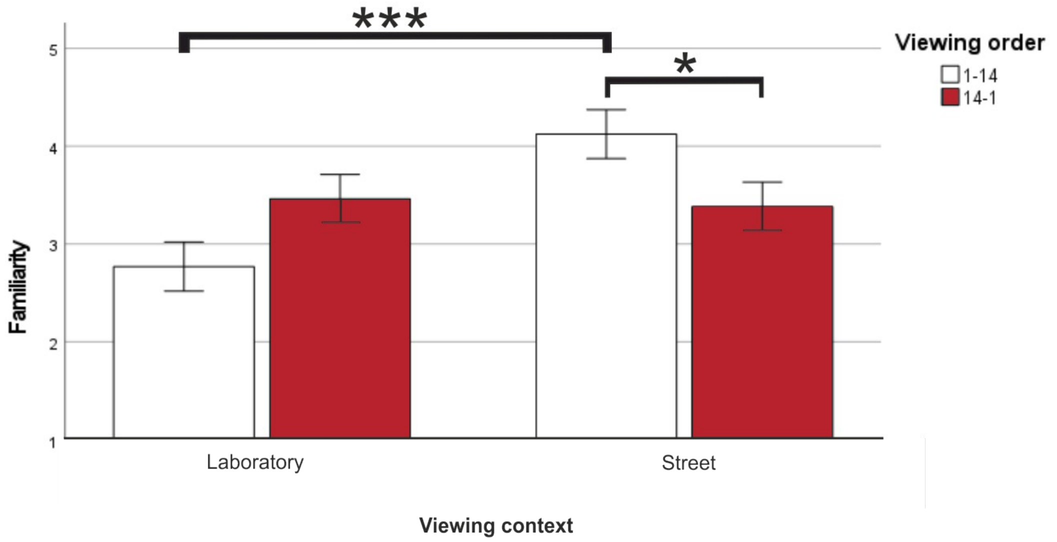


*Fig. S3*. Post hoc comparisons of the significant interaction effect of *viewing context* and *viewing order* on *familiarity with artworks*. Error bars represent ±1 standard error. * $p < .05$, *** $p < .001$.

**Viewers' Characteristics as Predictors of Art Appreciation**

Prediction of art appreciation from viewers' personal characteristics was explored through three stepwise multiple regression analyses. Respectively, *art identification* (Table S7), *liking* (Table S8), and *understanding* (Table S9) were regressed on expertise in the visual arts, interest in contemporary art, and need for closure.

Art identification could be positively predicted by interest in contemporary art alone, with an explained variance of 16%. Liking could be positively predicted by expertise in the visual arts alone, with an explained variance of 11%. Understanding could be positively predicted by interest in contemporary art alone, with an explained variance of 15%. There were no further significant models for all.

Table S7

*Multiple Regression of Art Identification*

| | Model 1 | |
|---|---|---|
| | $F(1, 84) = 16.81$, $p < .001$, $R^2_{\text{adj}} = .157$ | |
| | β | *p* |
| *Predictors* | | |
| Interest in Contemporary Art | .41 | < .001 |
| *Variables Excluded* | | |
| Need for Closure | .06 | .536 |
| Expertise in the Visual Arts | .14 | .227 |

Table S8

*Multiple Regression of Liking*

| | Model 1 | |
|---|---|---|
| | $F(1, 84) = 11.90$, $p < .001$, $R^2_{adj} = .114$ | |
| | β | *p* |
| *Predictors* | | |
| Expertise in the Visual Arts | .35 | < .001 |
| *Variables Excluded* | | |
| Need for Closure | .07 | .536 |
| Interest in Contemporary Art | .21 | .087 |

Table S9

*Multiple Regression of Understanding*

| | Model 1 | |
|---|---|---|
| | $F(1, 84) = 15.53$, $p < .001$, $R^2_{adj} = .146$ | |
| | β | *p* |
| *Predictors* | | |
| Interest in Contemporary Art | .40 | < .001 |
| *Variables Excluded* | | |
| Need for Closure | .03 | .743 |
| Expertise in the Visual Arts | .19 | .119 |
